# Color Image Encryption Based on an Evolutionary Codebook and Chaotic Systems

**DOI:** 10.3390/e26070597

**Published:** 2024-07-12

**Authors:** Yuan Cao, Yinglei Song

**Affiliations:** School of Automation, Jiangsu University of Science and Technology, Zhenjiang 212003, China; 221110303105@stu.just.edu.cn

**Keywords:** color images, image encryption, bit-level operations, evolutionary codebook, chaotic systems

## Abstract

Encryption of images is an important method that can effectively improve the security and privacy of crucial image data. Existing methods generally encrypt an image with a combination of scrambling and encoding operations. Currently, many applications require highly secure results for image encryption. New methods that can achieve improved randomness for both the scrambling and encoding processes in encryption are thus needed to further enhance the security of a cipher image. This paper proposes a new method that can securely encrypt color images. As the first step of the proposed method, a complete bit-level operation is utilized to scramble the binary bits in a color image to a full extent. For the second step, the bits in the scrambled image are processed with a sweeping operation to improve the encryption security. In the final step of encryption, a codebook that varies with evolutionary operations based on several chaotic systems is utilized to encrypt the partially encrypted image obtained in the second step. Experimental results on benchmark color images suggest that this new approach can securely encrypt color images and generate cipher images that remain secure under different types of attacks. The proposed approach is compared with several other state-of-the-art encryption approaches and the results show that it can achieve improved encryption security for cipher images. Experimental results thus suggest that this new approach can possibly be utilized practically in applications where color images need to be encrypted for content protection.

## 1. Introduction

Nowadays, images have become an important form of digital data that can accurately store a large amount of information. The contents in many images contain crucial information and thus need to be made available only to certain groups of individuals. The security of such images is generally improved by algorithms designed to encrypt images [1,2]. To achieve satisfactory security levels for cipher images, numerous computational methods have been developed for the encryption of images [3,4,5,6,7]. So far, three important types of computational methods have been developed for image encryption, including approaches that perform encryption by pixel permutations [3,8], methods that encrypt images by transformations of pixel values [4,9,10], and encryption techniques based on chaotic systems [5,11].

For methods based on pixel permutations, the pixels in an image are relocated to new positions for encryption. For instance, the work in [12] encrypts an image by changing pixel locations based on ergodic matrices. In [13], the positions of pixels are changed with Peano–Hilbert curves to significantly reduce the spatial correlations among them. In [14], images are encrypted by combining a variety of permutation techniques with edge maps constructed from source images. In [8], locations and pixel values are both changed to process a plain image to generate its cipher image. The work in [3] utilizes a permutation–diffusion architecture together with a skew tent map system to encrypt images. In [15], an elliptic curve random generator is employed to significantly enhance the security of cipher images.

Another method applies transformations to pixel values in an image to perform its encryption. For example, the gyrator transform is utilized together with the Arnold transform to generate cipher images [16]. In [9], the Arnold transform is combined with the discrete fractional random transform (DFRNT) to encrypt color images. In [17], deoxyribonucleic acid (DNA) encoding is used along with elliptic curve cryptography (ECC) to obtain cipher images with improved security. In [18], images are encrypted by a method that integrates compressive sensing with information hiding. The work in [19] proposes a novel type of discrete fractional Fourier transforms (DFrFs) based on a random-matrix scheme for the encryption of images. In [10], encryption of an image is performed by changing the pixel values in the image by the fractional Fourier transform (FRFT). The work in [20] encrypts color images based on a method that utilizes a Mandelbrot set along with DNA sequence operations and the Arnold map. In [4], images are iteratively encrypted with an approach that uses random phase encoding and gyrator transform. The work in [21] develops an approach that can encrypt images with a constructed nonlinear S-box. In [22], images are adaptively encrypted by using a self-adaptive permutation–diffusion approach together with DNA random encoding. The work in [23] shows that DNA coding and compressive sensing can be combined to generate cipher images with significantly improved security.

Recently, chaotic systems have provided an important tool that can securely encrypt images [5,7]. Specifically, due to the fact that chaotic systems can generate outputs that are sensitive to their inputs to a high degree, cipher images with high security can be obtained by processing plain images with information generated from one or several chaotic systems. In [24], a piecewise linear chaotic transformation is utilized for the construction of a pseudo-random key stream sequence to encrypt an image. In [25], a system based on a neotype chaotic product trigonometric map (PTM) is proposed for the encryption of images. In [26], an image is encrypted by modifying the gray values and locations of pixels in the image based on several one-dimensional logistic systems. In [6], a plain image is processed with the information generated from a few chaotic systems to change the locations of its pixels for its encryption. In [27], a particle swarm optimization method is used along with chaotic systems to improve the security of cipher images. The approach proposed in [11] encrypts images with a high-dimensional chaotic system and several sequences of pseudo-random numbers obtained based on a simple perception. In [28], a few chaotic systems are utilized to control an evolutionary process that can encrypt color images.

Since the randomness associated with the outputs of chaotic systems generally leads to improved security for cipher images, methods that can effectively combine chaotic systems with other encryption techniques have been proposed for image encryption [20,29,30,31,32]. For instance, a method that utilizes both fractal sorting matrices (FSMs) and global chaotic pixel diffusion is proposed in [32] to encrypt images. In [29], DNA sequence operations are effectively combined with chaotic systems to process a plain image for its encryption. The method developed in [33] constructs a two-dimensional logistic tent modular map (2D-LTMM) to improve the security of a cipher image. In [34], a secure hash algorithm (SHA), SHA-2, is utilized with DNA operations and hyper-chaotic systems to encrypt images. The method proposed in [35] integrates a chaotic system that contains a hidden attractor with the Knuth–Durstenfeld algorithm to enhance encryption security. In [36], DNA sequence operations are used with hyper-chaotic systems for image fusion encryption. The work in [37] encrypts images using the information obtained based on the mixed linear–nonlinear coupled map lattices (MLNCMLs). The work in [38] proposes an approach that employs DNA sequence operations and chaotic systems to significantly enhance encryption security. In [39], the security of cipher images can be enhanced by the integration of a permutation–substitution (SP) network with chaotic systems. In [31], the security of cipher images is improved by using DNA operations with a 5D hyper chaotic system for encryption.

Although most of the computational methods that have been proposed for image encryption can achieve excellent performance on encryption security, new encryption approaches that can further enhance the security of encryption are highly desirable to further upgrade the security level of cipher images. Encryption security can potentially be improved in two aspects. Firstly, the scrambling process utilized to eliminate the local correlations that usually exist among pixels in a plain image can be performed with new bit-level operations to completely remove the information in a plain image. In addition, the encryption security can possibly be further improved by applying an additional bit-level transformation to the bits in a scrambled image. Secondly, transformations that can enhance the randomness of a cipher image can be utilized to further improve the security of encryption.

In this paper, a new method is proposed to securely encrypt color images using an evolutionary codebook determined based on the information generated by a number of chaotic systems. The proposed method encrypts a color image in three phases. In the first phase, binary bits that represent the values of pixels in a plain image are partitioned into a few sequences with a number of mappings generated from a one-dimensional logistic system. The binary bits in each sequence are placed into a rectangular structure constructed from the output of a second one-dimensional logistic system. Each bit in the rectangular structure is relocated to a new position in the structure based on a set of operations performed on each row and column in the structure. The bits in the rectangular structure are mapped back to the sequence based on the transformation utilized to construct the rectangular structure. The scrambling process is complete when the bits in each sequence have been relocated to new positions in the sequence. In the second phase, a sequence of bit exclusive-or operations is accumulatively applied to the bits in each column and row of a scrambled image to further improve the plain image sensitivity of a cipher image. A partially encrypted image can be obtained as a result. In the third phase, the binary bits in a partially encrypted image are sequentially divided into several subsequences with the same length. All subsequences are encoded sequentially based on an evolutionary codebook generated from several chaotic systems. After the encoding of a subsequence is performed, the codebook is updated by a cross-over operation and a mutation operation determined by the outputs of the chaotic systems. A cipher image can be obtained by combining the encoded sequences of all subsequences in the partially encrypted image.

A summary of the major contributions of the paper can be listed as follows.

1. A new approach that can fully scramble the bits in a plain image and significantly reduce the local correlations that usually exist among pixels in the image is proposed.
2. A bit-level sweeping approach that can improve the plain image sensitivity of a cipher image is proposed.
3. A novel method that can securely encrypt the values of pixels in a scrambled image with a codebook constructed and varied using a number of chaotic systems is proposed.

Estimation suggests that the proposed method has a key space that is large in size, which indicates that attacks based on an exhaustive search cannot decipher a cipher image obtained using the proposed method. For testing, a number of benchmark color images and color images selected from the BSD dataset [40] are encrypted with the proposed method. Evaluations based on several different measures of security show that the proposed approach is capable of generating cipher images with high security. A comparison of the proposed approach with a few other state-of-the-art methods for encryption suggests that this new approach can provide cipher images that have improved overall security.

## 2. Materials and Methods

The proposed approach processes a plain color image in three phases to obtain its cipher image. For the first phase, the binary bits that represent the values of pixels in the plain image are partitioned into sequences and the bits in each sequence are scrambled with a rectangular structure constructed based on a one-dimensional logistic system. The scrambled sequences are then combined into a scrambled image. In the second phase, the bits in each row of a scrambled image are accumulatively processed by a sequence of exclusive-or bit operations, followed by a set of exclusive-or bit operations accumulatively applied to the bits in each column of the scrambled image. A partially encrypted image is generated as a result. In the third phase, the binary bits in a partially encrypted image are grouped into subsequences, and all subsequences are sequentially encoded with an evolutionary codebook. After a subsequence is encoded, the codebook is modified by a cross-over operation and a mutation operation determined from the information generated by a number of chaotic systems. A cipher image is obtained when the encoding of all subsequences is complete.

**Figure 1:**
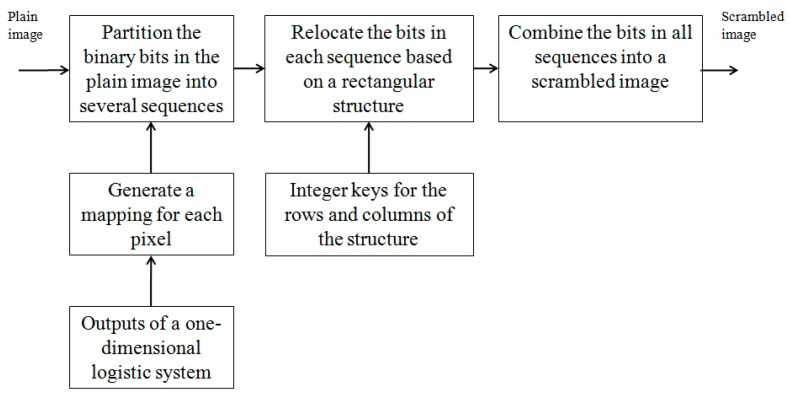
A description of the first phase of the proposed approach for generating a scrambled image.

Figure 1 provides a description of the major steps of the first phase of the proposed approach. From the information generated by a one-dimensional logistic system, a mapping is generated for each pixel in a plain image. Based on these mappings, the binary bits that represent the values of pixels in the plain image are partitioned into several sequences of bits. For each sequence, a rectangular structure is constructed from the output of another logistic system, and the bits in the sequence are sequentially mapped into the rectangular structure. The bits in each row of the rectangular structure are relocated to new positions in the same row based on a positive integer key that is associated with the row. Similarly, the locations of the bits in each column of the structure are changed to new values using a map constructed from the integer key for the column. After the relocations of all bits are performed, the bits in the rectangular structure are mapped back to the sequence to complete the scrambling operation on the sequence. A scrambled image is obtained after the scrambling operations on all sequences have been performed.

**Figure 2:**
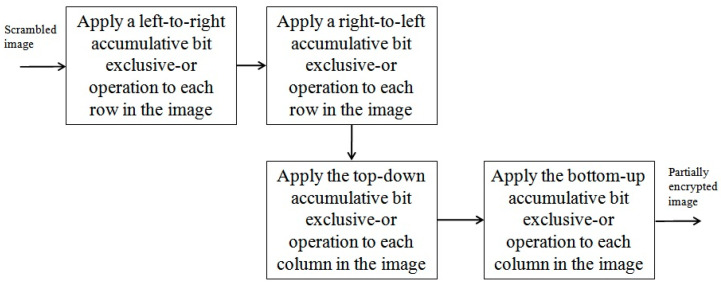
A description of the sweeping process in the second phase of the proposed approach.

The major steps that implement the second phase of the proposed approach are shown in Figure 2. Firstly, for each row in a scrambled image, a left-to-right sweeping operation is performed on all bits in the row such that each bit is replaced by the exclusive-or of all bits to the left of the bit and itself. A similar right-to-left sweeping operation is then applied to all bits in the same row, where each bit is replaced by the exclusive-or of all bits to the right of the bit and itself. Secondly, for each column in a scrambled image, all bits in the column are processed such that each bit is replaced by the exclusive-or of all bits above the bit and itself. All bits in the same column are then processed in a bottom-up order, where each bit is replaced by the exclusive-or of all bits below the bit and itself. A partially encrypted image is obtained after the operations on all rows and columns are complete.

**Figure 3:**
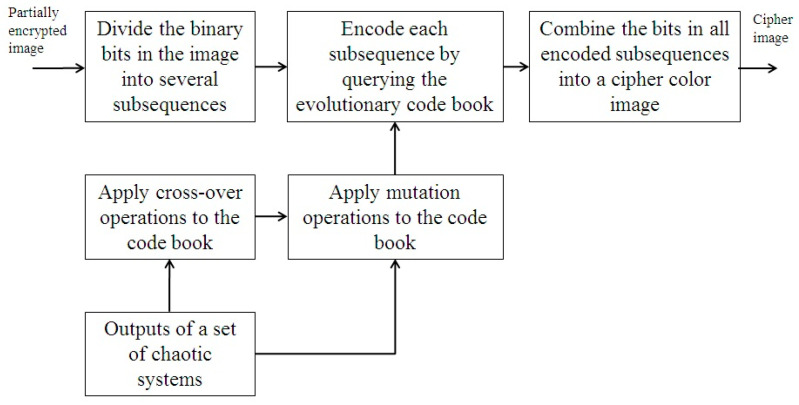
A description of the encoding process in the third phase of the proposed approach.

Figure 3 illustrates the major steps in the third phase of the proposed approach. For encoding, the binary bits in a partially encrypted image are sequentially divided into a number of subsequences of a given length. Each subsequence is encoded with a codebook constructed from the information generated by a number of chaotic systems. After a subsequence is encoded, the entries in the codebook are relocated to new positions based on a cross-over operation and a mutation operation determined by the information generated by the chaotic systems. For the cross-over operation, an integer key obtained from the output of a chaotic system is utilized to partition the codebook into two different parts, the entries in each part are then relocated to new positions based on the outputs of two other chaotic systems. The entries in both parts are combined into a new codebook. For the mutation operation, each entry in a codebook is moved to a new position in the codebook using a mapping based on the output of another chaotic system. A cipher image is generated after all subsequences are sequentially encoded with the evolutionary codebook.

A cipher image can be decrypted in three major phases. In the first phase of decryption, the binary bits that represent the values of pixels in the cipher image are sequentially divided into subsequences based on the length of the subsequences used in encoding. The evolutionary codebook used to encode the pixel values in a partially encrypted image is sequentially constructed and searched to determine the decrypted subsequence for each subsequence in the cipher image. A partially decrypted image is obtained by combining the decrypted subsequences into a color image.

In the second phase of decryption, the bits in each column of the partially decrypted image are first processed based on a sequence of accumulative bit exclusive-or operations. Specifically, each bit in the column is first replaced by the exclusive-or of all bits below it and itself. After the operation is complete, each bit in the column is replaced by the exclusive-or of all bits above it and itself. Each row is processed similarly after all columns are processed. Each bit in a row is first replaced by the exclusive-or of all bits to the right of the bit and itself. After the operation is complete, each bit in the row is replaced by the exclusive-or of all bits to the left of the bit and itself. A scrambled image is obtained as a result.

In the third phase of decryption, the binary bits in the scrambled image are partitioned into several sequences based on the mappings used to partition the plain image in the scrambling process. For each sequence, the rectangular structure used to relocate the bits in the sequence for scrambling is constructed and the mapped position for each bit location in the sequence is obtained based on the scrambling operations performed in each row and column of the rectangular structure. An array can be maintained to store the mapped position for each bit location in the sequence. Based on the array, each bit in the sequence can be moved to its original position in the plain image. The plain image of the cipher image is recovered when the bits in all sequences have been moved to their original positions in the plain image.

### 2.1. The Scrambling of Bits

Let P be a plain color image that contains h rows and w columns, P(i,j, 1), $P\left(i,j,2\right)$, and $P\left(i,j,3\right)$ represent the R, G, and B components of the pixel in row i and column j of P, respectively, where 1≤i≤h and 1≤j≤w hold for integers i and j. Since the R, G, and B components associated with a pixel contain 24 bits in total, the binary bits in P are partitioned into 24 different sequences based on the outputs of a one-dimensional logistic system $L_{1}$.

A one-dimensional logistic system can generate a real number sequence $x_{1},x_{2},\ldots ,x_{n}\ldots$ from an initial value $x_{0}$ that satisfies $0<x_{0}<1$. For integer k≥0, the recursion relation between $x_{k}$ and $x_{k+1}$ is shown in Equation (1).

(1) $$x_{k+1}=4x_{k}(1-x_{k})$$

It has been shown that the one-dimensional logistic system defined in Equation (1) is chaotic if there does not exist a real number $\theta_{0}$ such that $x_{0}=\sin^{2}\theta_{0}$ and $\theta_{0}=h_{d}\pi /(2^{d_{1}}-2^{d_{2}})$ hold, where $h_{d}$, $d_{1}$, $d_{2}$ are integers that satisfy $h_{d}>0$ and $d_{1}>d_{2}\geq 0$ [41]. In the rest of the paper, it is assumed that all initial values selected for Equation (1) lead to chaotic one-dimensional logistic systems. An initial value slightly different from $x_{0}$ would lead to a significantly different value for $x_{k}$ when k is sufficiently large. Let N be a positive integer, $y_{0}$ and $z_{0}$ be the initial values of $L_{1}$, $y_{1},y_{2},\ldots ,y_{N},\ldots$ and $z_{1},z_{2},\ldots ,z_{N},\ldots$ be the sequences of real numbers generated from $y_{0}$ and $z_{0}$ with Equation (1), respectively. From Equation (2), integer X  can be obtained with a large positive integer $N_{0}$.

(2) $$X=\left\lfloor y_{N}\times N_{0}\right\rfloor$$

Let $c_{1},c_{2},\ldots ,c_{hw}$ be the first hw integers that are larger than X and coprime with 24, Based on Equation (3) and $N_{0}$, let $r\left(i,j\right)=\left(i-1\right)w+j$, integer $b_{r(i,j)}$ is generated for the pixel that is located in column j and row i of P.

(3) $$b_{r(i,j)}=\left\lfloor z_{r(i,j)+N}\times N_{0}\right\rfloor \;mod\;24$$

A mapping $\sigma_{r(i,j)}$ is constructed for the pixel in row i and column j of P. For an integer s that satisfies 1≤s≤24, $\sigma_{r(i,j)}(s)$ is the mapped integer for s based on Equation (4).

(4) $$\sigma_{r(i,j)}\left(s\right)=\left(c_{r(i,j)}(s-1)+b_{r(i,j)}\right)\;mod\;24+1$$

It is clear from Equation (4) that $1\leq \sigma_{r(i,j)}(s)\leq 24$ holds and $\sigma_{r(i,j)}(s_{1})$ and $\sigma_{r(i,j)}(s_{2})$ are different for two different integers $s_{1}$ and $s_{2}$ that satisfy $1\leq s_{1}\leq 24$ and $1\leq s_{2}\leq 24$. Otherwise, the following equation must hold for $s_{1}$ and $s_{2}$, where $k_{0}$ is a positive integer.

(5) $$c_{r(i,j)}\left|s_{1}-s_{2}\right|=24k_{0}$$

From Equation (5), since $c_{r(i,j)}$ and 24 are coprime, 24 must be an integer factor of $\left|s_{1}-s_{2}\right|$. This contradicts the fact that $1\leq \left|s_{1}-s_{2}\right|<24$ holds, $\sigma_{r(i,j)}(s_{1})$ and $\sigma_{r\left(i,j\right)}\left(s_{2}\right)$ thus must be different. In other words, $\sigma_{r(i,j)}$ is a one-to-one mapping for integers between 1 and 24.

The binary bits that represent the values of all pixels in P can be partitioned into 24 different sequences based on the mappings that have been constructed for pixels in P. Specifically, an array of 24 bits can be constructed by combining the R, G, and B components of each pixel in P. Let $F_{r(i,j)}$ be the array of binary bits for the pixel in column j and row i in P. The first, second, and third 8 bits in $F_{r(i,j)}$ are binary representations for the R, G, and B components in the pixel value. $F_{r(i,j)}\left(k\right)$ denotes the bit in the kth location in $F_{r(i,j)}$. Given an integer k such that 1≤k≤24 holds, for each integer t that satisfies 1≤t<hw, Equations (6) and (7) recursively define a mapping $\mu_{t}$.

(6) $$\mu_{1}\left(k\right)=\sigma_{1}(k)$$

(7) $$\mu_{t+1}\left(k\right)=\sigma_{t}(\mu_{t}\left(k\right))$$

For each k that satisfies 1≤k≤24, a sequence $S_{k}$ of hw bits can be formed such that the bit in position t of $S_{k}$ is set to be $F_{t}(\mu_{t}\left(k\right))$ for each positive integer t such that 1≤t≤hw holds. Since $\sigma_{t}$ is a one-to-one mapping, $S_{1},\;S_{2},\ldots ,S_{24}$ is a disjoint partition of the bits in the pixel values of P.

For each k that satisfies 1≤k≤24, the binary bits in $S_{k}$ are relocated for scrambling at the bit level. Let $S_{k}(l)$ be the bit in position l, where 1≤l≤hw. The relocation of bits in $S_{k}$ is performed based on a rectangular structure $R_{k}$. For a real number $0<l_{0}<1$, a sequence of real numbers $l_{1},l_{2},\ldots ,l_{N},\ldots$ is generated using Equation (1). The number of rows $r_{k}$ in $R_{k}$ is determined from $l_{N+f(k)}$ as shown in Equation (8), where f(k) is an integer function of k and its definition is provided later in the paper.

(8) $$r_{k}=\left(\left\lfloor l_{N+f(k)}\times N_{0}\right\rfloor -1\right)\;mod\;h+1$$

The number of columns $u_{k}$ in $R_{k}$ is obtained from $r_{k}$ as shown in Equation (9).

(9) $$u_{k}=\left\lceil \frac{hw}{r_{k}}\right\rceil$$

For each integer l that satisfies 1≤l≤hw, $S_{k}(l)$ is placed into a location in $R_{k}$ based on the mappings described by Equations (10) and (11), where $p_{l}$ is the row number of the location in $R_{k}$ and $q_{l}$ is its column number in $R_{k}$.

(10) $$p_{l}=\left\lceil \frac{l}{u_{k}}\right\rceil$$

(11) $$q_{l}=\left(l-1\right)\;mod\;u_{k}+1$$

A position in $R_{k}$ that is not mapped to a bit in $S_{k}$ is assigned a value of −1. Each row and column in $R_{k}$ is associated with a positive integer key. Let $s_{1},s_{2},\ldots s_{r_{k}}$ be the first $r_{k}$ integers that are larger than $\left\lfloor l_{N+f(k)}\times N\right\rfloor$ and coprime with $u_{k}$. The integer key for row m in $R_{k}$ is $s_{m}$. In addition, an integer $v_{m}$ is determined for row m as shown in Equation (12).

(12) $$v_{m}=\left(\left\lfloor l_{N+f\left(k\right)+m}\times N_{0}\right\rfloor -1\right)\;mod\;u_{k}+1$$

The scrambling process first changes the positions of bits in each row of $R_{k}$ to new positions in the same row. The bit in position d of row m is relocated to position $f_{d}$ in the same row. $f_{d}$ is determined from d as shown in Equation (13).

(13) $$f_{d}=\left(\left(d-1\right)s_{m}+v_{m}\right)\;mod\;u_{k}+1$$

Since $s_{m}$ and $u_{k}$ are coprime, no two different positions in the same row are relocated to the same position.

After the scrambling of all rows in $R_{k}$ are complete, the bits in each column of $R_{k}$ are moved to new positions in the same column. Let $g_{1},g_{2},\ldots g_{u_{k}}$ be the first $u_{k}$ integers that are larger than $s_{r_{k}}$ and coprime with $r_{k}$. $g_{n}$ is the integer key for column n. The bit in position e of column n is relocated to position $h_{e}$ in the same column. $h_{e}$ is obtained from e with Equation (14), where $w_{n}$ is obtained for column n based on Equation (15).

(14) $$h_{e}=\left(\left(e-1\right)g_{n}+w_{n}\right)\;mod\;r_{k}+1$$

(15) $$w_{n}=\left(\left\lfloor z_{N+hw+e}\times N_{0}\right\rfloor -1\right)\;mod\;r_{k}+1$$

Since $g_{n}$ is coprime with $r_{k}$, no two different positions in the same column are relocated to the same position. Function f(k) in Equation (12) can be recursively defined based on Equations (16) and (17).

(16) $$f\left(1\right)=0$$

(17) $$f\left(k\right)=f\left(k-1\right)+u_{k}+1$$

After the scrambling operations on all columns in $R_{k}$ are completely performed, the bits in $R_{k}$ are mapped to the corresponding positions in $S_{k}$. A sequence $T_{k}$ that contains $r_{k}u_{k}$ bits and values is constructed. For the position in row $i_{r}$ and column $j_{r}$ of $R_{k}$, let $m_{r}(i_{r},j_{r})$ be its corresponding location in $T_{k}$. $m_{r}(i_{r},j_{r})$ is determined from Equation (18) as follows.

(18) $$m_{r}\left(i_{r},j_{r}\right)=\left(i_{r}-1\right)u_{k}+j_{r}$$

The bit or value in row $i_{r}$ and column $j_{r}$ of $R_{k}$ is thus assigned to position $m_{r}(i_{r},j_{r})$ in $T_{k}$. The scrambled sequence for $S_{k}$ can be obtained from $T_{k}$ by eliminating the positions that contain a value of −1 from $T_{k}$.

A scrambled image D can be obtained from the scrambled sequences for $S_{1},S_{2},\ldots ,S_{24}$. Specifically, for each pair of integers (k,t) that satisfies 1≤k≤24 and 1≤t≤hw, set $F_{t}(\mu_{t}\left(k\right))$ to be the bit in position t of the scrambled sequence for $S_{k}$. The resulting image is a scrambled image D for P.

### 2.2. The Sweeping Operations

A partially encrypted image can be obtained by accumulatively applying a set of bit exclusive-or operations to the bits in a scrambled image. The operations are performed on the bits in each row of the scrambled image first. After the operations on all rows are complete, similar operations are applied to each column in the scrambled image. As a result, a partially encrypted image is obtained after the sweeping operation is complete.

Given a bit value $b_{0}$ and an array A of $s_{z}$ binary bits, a sweeping operation $A_{s}=SW(b_{0},A)$ generates a new array $A_{s}$ of the same size as that of A. Let $p_{z}$ be an integer that satisfies $1<p_{z}\leq s_{z}$, $A(p_{z})$ and $A_{s}(p_{z})$ are the bits in position $p_{z}$ of A and $A_{s}$, respectively. $A_{s}\left(p_{z}\right)$ can be recursively obtained based on bit exclusive-or operations with Equations (19) and (20).

(19) $$A_{s}\left(1\right)=A(1)⊕b_{0}$$

(20) $$A_{s}\left(p_{z}\right)=A_{s}\left(p_{z}-1\right)⊕A(p_{z})$$

For row $i_{r}$ in D, where $i_{r}$ is an integer that satisfies $1\leq i_{r}\leq h$, a left-to-right sweeping operation on row $i_{r}$ is performed by sequentially applying a sweeping operation to the array of bits of all pixels in row $i_{r}$ from left to right. The operations are described in Equations (21) and (22), where $b_{r}=\left(i_{r}-1\right)w$ and k is a positive integer such that 1<k≤w holds.

(21) $$V_{l,1}=SW(0,F_{b_{r}+1})$$

(22) $$F_{l,k}=SW(V_{l,k-1}\left(24\right),\;F_{b_{r}+k})$$

After $V_{l,1},\;V_{l,2},\ldots V_{l,w}$ are obtained, the bits in $F_{b_{r}+k}$ are set to be those in $V_{l,k}$ for each k. A right-to-left sweeping operation is then applied to the pixels in row $i_{r}$ based on Equations (23) and (24).

(23) $$K_{l,w}=SW(0,F_{b_{r}+w})$$

(24) $$K_{l,k-1}=SW(K_{l,k}\left(24\right),\;F_{b_{r}+k-1})$$

After $K_{l,1},\;K_{l,2},\ldots K_{l,w}$ are determined, the bits in $F_{b_{r}+k}$ are reset to be those in $K_{l,k}$ for each k.

After each row in D has been processed by the sweeping operation, similar sweeping operations are performed on the pixels in each column of D. Let $j_{c}$ be an integer that satisfies $1\leq j_{c}\leq w$, the pixels in column $j_{c}$ are sequentially processed with a top-down sweeping operation described by Equations (25) and (26), where k is a positive integer such that 1≤k≤h holds.

(25) $$H_{c,1}=SW(0,F_{j_{c}})$$

(26) $$H_{c,k}=SW(H_{c,k-1}\left(24\right),\;F_{\left(k-1\right)w+j_{c}})$$

After $H_{c,1},\;H_{c,2},\ldots H_{c,h}$ are obtained, the bits in $F_{\left(k-1\right)w+j_{c}}$ are set to be those in $H_{c,k}$ for each k. A bottom-up sweeping operation is then utilized to process the pixels in column $j_{c}$ with Equations (27) and (28).

(27) $$T_{c,h}=SW(0,F_{\left(h-1\right)w+j_{c}})$$

(28) $$T_{c,k-1}=SW(T_{c,k}\left(24\right),\;F_{(k-2)w+j_{c}})$$

After $T_{c,1},\;T_{c,2},\ldots T_{c,h}$ are determined, the bits in $T_{c,k}$ are assigned to $F_{\left(k-1\right)w+j_{c}}$ for each k. A partially encrypted image $E_{p}$ is obtained when the sweeping operations have been applied to all columns in D.

### 2.3. The Encoding of Pixels

The encoding of pixels in a partially encrypted image $E_{p}$ is performed with the information generated by a number of one-dimensional chaotic systems based on Equation (29), where k≥0 is an integer.

(29) $$x_{k+1}=(3\left(2x_{k}-1\right)-4{\left(2x_{k}-1\right)}^{3}+1)/2$$

The system described in Equation (29) is chaotic when the initial value $x_{0}$ satisfies $0<x_{0}<1$ and there does not exist a real number α such that $x_{0}=(1+\sin\alpha )/2$ and $\alpha ={2k}_{d}\pi /(3^{d_{1}}-3^{d_{2}})$ hold, where $k_{d},d_{1},d_{2}$ are integers and $d_{1}$, $d_{2}$ satisfy $d_{1}>d_{2}\geq 0$. Indeed, since $0<x_{0}<1$ holds for $x_{0}$, there exists a real number $\theta_{0}$ such that $-\pi /2<\theta_{0}<\pi /2\;$ and $x_{0}=(1+\sin\theta_{0})/2$. It is straightforward to see that Equation (30) holds for $x_{k}$.

(30) $$x_{k}=(1+\sin{(3}^{k}\theta_{0}))/2$$

Since $x_{0}=(1+\sin\theta_{0})/2$, the equation holds when k=0. Now, if Equation (30) holds when k=l, from Equation (29), $x_{l+1}$ can be obtained by $x_{l+1}=(3\sin\left(3^{l}\theta_{0}\right)-4{(\sin\left(3^{l}\theta_{0}\right))}^{3}+1)/2$, it is thus clear that $x_{l+1}=(1+\sin(3^{l+1}\theta_{0}))/2$, the equation holds for k=l+1. From the principle of induction, Equation (30) holds for all integers k≥0. Since $-\pi /2<\theta_{0}<\pi /2$ and there are no existing integers $k_{d},d_{1},d_{2}$ such that $\theta_{0}={2k}_{d}\pi /(3^{d_{1}}-3^{d_{2}})$ and $d_{1}>d_{2}\geq 0$ hold, Equation (30) suggests that the system is chaotic and a tiny amount of perturbation in $x_{0}$ would lead to a significantly different value for $x_{k}$ when k is a positive integer that is sufficiently large. In the rest of the paper, all initial values for Equation (30) are chosen such that the corresponding systems are chaotic.

The bits in the pixel values of $E_{p}$ are sequentially divided into subsequences of length 4. Specifically, for positive integers i and j such that 1≤i≤h and 1≤j≤w hold, an array $F_{r(i,j)}$ of 24 bits can be obtained by combining the binary bits in the R, G, and B components of the pixel in column j and row i of $E_{p}$, where $r\left(i,j\right)=\left(i-1\right)w+j$. The bits in $F_{r(i,j)}$ are sequentially divided into 6 subsequences $Q_{6r\left(i,j\right)-5}$, $Q_{6r\left(i,j\right)-4}$, $Q_{6r\left(i,j\right)-3}$, $Q_{6r\left(i,j\right)-2}$, $Q_{6r\left(i,j\right)-1}$, and $Q_{6r\left(i,j\right)}$. Starting with subsequence $Q_{1}$, the subsequences are sequentially encoded. For each positive integer t such that 1≤t<6hw holds, $Q_{t+1}$ is encoded after the encoding of $Q_{t}$ is complete.

A codebook $C_{b,t\;}$ that contains the encoding of each possible binary sequence of length 4 is utilized to encode subsequence $Q_{t}$. $C_{b,t}$ contains 16 entries in total and can be queried based on the integer value of the subsequence that needs to be encoded. Let $v(Q_{t})$ be the integer value represented by $Q_{t}$, the entry in the position of $v\left(Q_{t}\right)+1$ in $C_{b,t\;}$ is the encoded subsequence for $Q_{t}$, such an entry is denoted by $C_{b,t}(v\left(Q_{t}\right)+1)$. $C_{b,t}$ is obtained from $C_{b,t-1}$ based on a cross-over operation and a mutation operation.$\;C_{b,0}$ is a codebook where the encoded subsequence for each subsequence is the subsequence itself.

#### 2.3.1. The Cross-Over Operation

A cross-over operation utilizes two integer keys ${1\leq K}_{p}<255$ and ${1\leq L}_{p}<255$ to partition a codebook C into two arrays $C_{0}$ and $C_{1}$. Specifically, an array $K_{a}$ of 16 bits is constructed by combining the 8-bit binary representations of $K_{p}$ and $L_{p}$. The first 8 bits in $K_{a}$ are the binary representation of $K_{p}$ and the remaining 8 bits are that of $L_{p}$. For each integer $i_{d}$ that satisfies $1\leq i_{d}\leq 16$, the entry at $C(i_{d})$ is included in $C_{0}$ if $K_{a}(i_{d})$ is bit 0; otherwise, the entry is included in $C_{1}$. The relative orders for the entries in $C_{0}$ and $C_{1}$ remain the same as their relative orders in C.

Let $s_{z,0}$ and $s_{z,1}$ be the sizes of $C_{0}$ and $C_{1}$, respectively. Two other integer keys $I_{0}$ and $I_{1}$ are utilized to change the locations of the entries in $C_{0}$ and $C_{1}$, respectively. $I_{0}$ is required to be coprime with $s_{z,0}$ and $I_{1}$ must be coprime with $s_{z,1}$. Each entry in $C_{0}$ is relocated to a new position in $C_{0}$. The entry in position $j_{d}$ of $C_{0}$ is relocated to a new location $m_{0,j_{d}}$ in $C_{0}$, where $1\leq m_{0,j_{d}}\leq s_{z,0}$ and Equation (31) holds for $m_{0,j_{d}}$, where $x_{j_{d}}$ is an integer.

(31) $$s_{z,0}x_{j_{d}}+I_{0}\left(m_{0,j_{d}}-1\right)=j_{d}$$

Indeed, since $s_{z,0}$ and $I_{0}$ are coprime, a well-known fact is that there exist two integers $x_{z}$ and $y_{z}$ such that $s_{z,0}x_{z}+I_{0}y_{z}=1$ holds. If $\alpha_{j_{d}}=\left\lfloor y_{z}j_{d}/s_{z,0}\right\rfloor$, $m_{0,j_{d}}=y_{z}j_{d}-\alpha_{j_{d}}s_{z,0}+1$, and $x_{j_{d}}=\alpha_{j_{d}}I_{0}+x_{z}j_{d}$, it is clear that $s_{z,0}x_{j_{d}}+I_{0}\left(m_{0,j_{d}}-1\right)=(s_{z,0}x_{z}+I_{0}y_{z}$)$j_{d}=j_{d}$. Therefore, there exists an integer solution $\left(x_{j_{d}},m_{0,j_{d}}\right)$ for Equation (31) and $1\leq m_{0,j_{d}}\leq s_{z,0}$ holds.

For a given integer $j_{d}$, there exists only one integer $1\leq m_{0,j_{d}}\leq s_{z,0}$ that can satisfy Equation (31). If this is not the case, two different integer solutions $\left(x_{j_{d},\;0},m_{0,j_{d}}\right)$ and $\left(x_{j_{d},\;1},m_{1,j_{d}}\right)$ exist for Equation (31) such that both $1\leq m_{0,j_{d}}\leq s_{z,0}$ and $1\leq m_{1,j_{d}}\leq s_{z,0}$ hold. $m_{0,j_{d}}$ and $m_{1,j_{d}}$ thus must satisfy the following equation.

(32) $$s_{z,0}\left|x_{j_{d},0}-x_{j_{d},1}\right|=I_{0}|m_{0,j_{d}}-m_{1,j_{d}}|$$

However, since $s_{z,0}$ and $I_{0}$ are coprime, $s_{z,0}$ must be an integer factor of $\left|m_{0,j_{d}}-m_{1,j_{d}}\right|$, which contradicts the fact that $1\leq \left|m_{0,j_{d}}-m_{1,j_{d}}\right|<s_{z,0}$. Therefore, there exists only one integer $1\leq m_{0,j_{d}}\leq s_{z,0}$ that can satisfy Equation (31) for a given integer $j_{d}$.

In addition, the integer solutions $m_{0,i_{d}}$ and $m_{0,j_{d}}$ for two different integers $1\leq i_{d}\leq s_{z,0}$ and $1\leq j_{d}\leq s_{z,0}$ are different. Otherwise, the following equation must hold for $i_{d}$ and $j_{d}$, where k is a positive integer.

(33) $$s_{z,0}k=|i_{d}-j_{d}|$$

Equation (33) contradicts the fact that $1\leq \left|i_{d}-j_{d}\right|<s_{z,0}$. $m_{0,i_{d}}$ and $m_{0,j_{d}}$ thus must be two different integers.

The above argument suggests that the mapping from $j_{d}$ to $m_{0,j_{d}}$ based on Equation (31) is a one-to-one mapping for each integer $j_{d}$ that satisfies $1\leq j_{d}\leq s_{z,0}$. Each entry in $C_{0}$ can thus be relocated to a new position in $C_{0}$ based on the mapping. Similarly, since $s_{z,1}$ is coprime with $I_{1}$, a similar mapping can be constructed for $C_{1}$ based on $I_{1}$ and each entry in $C_{1}$ can be relocated to a new position in $C_{1}$. After the relocation operations are completed for both $C_{0}$ and $C_{1}$, the entries in $C_{0}$ and $C_{1}$ are merged to construct a new codebook $C_{n}$ based on $K_{a}$. The entries in $C_{0}$ are sequentially placed into positions in $C_{n}$ that have a bit of 0 in $K_{a}$, and the entries in $C_{1}$ are sequentially placed into positions in $C_{n}$ that have a bit of 1 in $K_{a}$.

The cross-over operation applied to $C_{b,t-1}$ is determined by the outputs of several chaotic systems described in Equation (29). Let $x_{r,0}$, $y_{r,0}$, $z_{r,0}$, $w_{r,0}$, $x_{g,0}$, $y_{g,0}$, $z_{g,0}$, ${\;w}_{g,0}$, ${\;x}_{b,0}$, ${\;y}_{b,0}$, $z_{b,0}$, ${\;and\;w}_{b,0}$ be 12 positive real numbers less than 1 and none of them are equal to 1/2. Using them as the initial values, 12 different sequences of real numbers can be generated with the recursive relation in Equation (29). For an integer k>0, ${\;x}_{r,k}$, $\;y_{r,k}$, $z_{r,k}$, ${\;w}_{r,k}$, $\;x_{g,k}$, ${\;y}_{g,k}$, ${\;z}_{g,k}$, ${\;w}_{g,k}$, ${\;x}_{b,k}$, ${\;y}_{b,k}$, $z_{b,k}$, ${\;and\;w}_{b,k}$ are the kth number in the sequences, respectively.

In cases where t mod 6=1 or t mod 6=2, the integer keys associated with the cross-over operation on $C_{b,t-1}$ are obtained from $x_{r,N+\delta_{t}}$, $y_{r,N+\delta_{t}}$, $z_{r,N+\delta_{t}}$, $\;and\;w_{r,N+\delta_{t}}$ based on Equations (34)–(37), where $K_{0}$ is a positive integer and $\delta_{t}$ is determined based on Equation (38), where $\tau_{0}$ is a positive integer. $K_{p,r}$ and $L_{p,r}$ are the integer keys utilized to construct the array for the partition operation in the cross-over operation on $C_{b,t-1}$, $I_{0,r}$ is the first integer that is larger than $I_{p,r}$ and coprime with $s_{z,0}$, and $I_{1,r}$ is the first integer that is larger than $J_{p,r}$ and coprime with $s_{z,1}$.$I_{0,r}$ and $I_{1,r}$ are the keys used to relocate the entries in the two parts of $C_{b,t-1}$ for cross-over.

(34) $$K_{p,r}=(\left(\left\lfloor x_{r,N+\delta_{t}}\times N_{0}\right\rfloor \;mod\;K_{0}\right)\times (v\left(Q_{t-1}\right)+1))mod\;254+1$$

(35) $$L_{p,r}=((\left\lfloor y_{r,N+\delta_{t}}\times N_{0}\right\rfloor \;mod\;K_{0})\times (v\left(Q_{t-1}\right)+1))\;mod\;254+1$$

(36) $$I_{p,r}=(\left\lfloor z_{r,N+\delta_{t}}\times N_{0}\right\rfloor \;mod\;K_{0})\times (v\left(Q_{t-1}\right)+1)$$

(37) $$J_{p,r}=(\left\lfloor w_{r,N+\delta_{t}}\times N_{0}\right\rfloor \;mod\;K_{0})\times (v\left(Q_{t-1}\right)+1)$$

(38) $$\delta_{t}=(2\left\lfloor t/6\right\rfloor +t\;mod\;6-1)\tau_{0}$$

Similarly, in cases where t mod 6=3 or t mod 6=4, the integer keys used for the cross-over operation on $C_{b,t-1}$ are obtained from $x_{g,N+\gamma_{t}}$, ${\;y}_{g,N+\gamma_{t}}$, $z_{g,N+\gamma_{t}}$, ${\;and\;w}_{g,N+\gamma_{t}}$ based on Equations (39)–(42). $\gamma_{t}$ is determined based on Equation (43). $K_{p,g}$ and $L_{p,g}$ are the integer keys utilized to perform the partition on $C_{b,t-1}$ for cross-over. Let $I_{0,g}$ be the first integer that is larger than $I_{p,g}$ and coprime with $s_{z,0}$, and $I_{1,g}$ be the first integer that is larger than $J_{p,g}$ and coprime with $s_{z,1}$. $I_{0,g}$ and $I_{1,g}$ are the keys used to relocate the entries in the two parts of $C_{b,t-1}$ for cross-over.

(39) $$K_{p,g}=(\left(\left\lfloor x_{g,N+\gamma_{t}}\times N_{0}\right\rfloor \;mod\;K_{0}\right)\times (v\left(Q_{t-1}\right)+1))mod\;254+1$$

(40) $$L_{p,g}=((\left\lfloor y_{g,N+\gamma_{t}}\times N_{0}\right\rfloor \;mod\;K_{0})\times (v\left(Q_{t-1}\right)+1))\;mod\;254+1$$

(41) $$I_{p,g}=(\left\lfloor z_{g,N+\gamma_{t}}\times N_{0}\right\rfloor \;mod\;K_{0})\times (v\left(Q_{t-1}\right)+1)$$

(42) $$J_{p,g}=(\left\lfloor w_{g,N+\gamma_{t}}\times N_{0}\right\rfloor \;mod\;K_{0})\times (v\left(Q_{t-1}\right)+1)$$

(43) $$\gamma_{t}=(2\left\lfloor t/6\right\rfloor +t\;mod\;6-3)\tau_{0}$$

Finally, in cases where t mod 6=5 or t mod 6=0, the integer keys used for the cross-over operation on $C_{b,t-1}$ are obtained from $x_{b,N+\epsilon_{t}}$, ${\;y}_{b,N+\epsilon_{t}}$, $z_{b,N+\epsilon_{t}}$, ${\;and\;w}_{b,N+\epsilon_{t}}$ based on Equations (44)–(47). $\varepsilon_{t}$ is determined with Equation (48), where $h_{t}=0$ if t mod 6=5, and $h_{t}=1$ if t mod 6=0. $K_{p,b}$ and $L_{p,b}$ are the integer keys used to partition $C_{b,t-1}$ for cross-over. Let $I_{0,b}$ be the first integer that is larger than $I_{p,b}$ and coprime with $s_{z,0}$, and $I_{1,b}$ be the first integer that is larger than $J_{p,b}$ and coprime with $s_{z,1}$. $I_{0,b}$ and $I_{1,b}$ are the keys employed to perform the relocation of the entries in the two parts of $C_{b,t-1}$ for cross-over.

(44) $$K_{p,b}=(\left(\left\lfloor x_{b,N+\varepsilon_{t}}\times N_{0}\right\rfloor \;mod\;K_{0}\right)\times (v\left(Q_{t-1}\right)+1))mod\;254+1$$

(45) $$L_{p,b}=((\left\lfloor y_{b,N+\varepsilon_{t}}\times N_{0}\right\rfloor \;mod\;K_{0})\times (v\left(Q_{t-1}\right)+1))\;mod\;254+1$$

(46) $$I_{p,b}=(\left\lfloor z_{b,N+\varepsilon_{t}}\times N_{0}\right\rfloor \;mod\;K_{0})\times (v\left(Q_{t-1}\right)+1)$$

(47) $$J_{p,b}=(\left\lfloor w_{b,N+\varepsilon_{t}}\times N_{0}\right\rfloor \;mod\;K_{0})\times (v\left(Q_{t-1}\right)+1)$$

(48) $$\varepsilon_{t}=(2\left\lfloor t/6\right\rfloor -h_{t})\tau_{0}$$

The value of $v\left(Q_{0}\right)$ is set to be 0 when integer keys for the cross-over operation on $C_{b,0}$ are computed. As a result of the cross-over operation on $C_{b,t-1}$, a new codebook $T_{b,t-1}$ can be obtained. $C_{b,t}$ is generated by applying a mutation operation to $T_{b,t-1}$.

#### 2.3.2. The Mutation Operation

A mutation operation changes a codebook $T_{b,t-1}$ in two steps. In the first step, the order of the entries in $T_{b,t-1}$ is altered with an approach similar to the one used in a cross-over operation. In the second step, all entries in $T_{b,t-1}$ are circularly shifted by a given number of positions.

A positive integer key $J_{0}$ is needed to perform the mutation operation on $T_{b,t-1}$. $J_{0}$ is required to be coprime with 16. For each integer $1\leq i_{m}\leq 16$, the entry in position $i_{m}$ of $T_{b,t-1}$ is relocated to position $n_{i_{m}}$. The integer $1\leq n_{i_{m}}\leq 16$ satisfies Equation (49), where $y_{i_{m}}$ is an integer.

(49) $$16y_{i_{m}}+J_{0}n_{i_{m}}=i_{m}$$

Since $J_{0}$ and 16 are coprime, from an argument that is similar to the one in Section 2.3.1, the mapping from $i_{m}$ to $n_{i_{m}}$ is a one-to-one mapping. Each entry in $T_{b,t-1}$ is relocated to a new position based on the mapping.

After all entries in $T_{b,t-1}$ have been relocated based on the mapping determined by Equation (49), each entry is circularly shifted by $s_{h}$ positions, where ${\;s}_{h}$ is determined based on Equation (50).

(50) $$s_{h}=J_{0}\;mod\;16$$

The entry in position $j_{m}$ of $T_{c,b}$ is shifted to position $l_{j_{m}}$ as described in Equation (51). The resulting codebook is $C_{b,t}$.

(51) $$l_{j_{m}}=\left(j_{m}+s_{h}\right)\;mod\;16+1$$

The integer key used to perform the mutation operation on $T_{b,t-1}$ is obtained from the information generated by the three chaotic systems defined in Equation (29). Let $u_{r,0}$, ${\;u}_{g,0}$, and $u_{b,0}$ be the initial values of the three chaotic systems. Three sequences of real numbers can be generated from $u_{r,0}$, ${\;u}_{g,0}$, and $u_{b,0}$. For a positive integer k, $u_{r,k}$, $\;u_{g,k}$, and $u_{b,k}$ are the kth real numbers of the three sequences, respectively.

For a positive integer t that satisfies 1≤t≤hw, when t mod 6=1 or t mod 6=2 holds. The integer key $J_{r,0}$ for the mutation operation on $T_{b,t-1}$ is the first integer that is larger than $H_{p,r}$ and coprime with 16. $H_{p,r}$ is determined with Equation (52), where $\delta_{t}$ is defined based on Equation (38).

(52) $$H_{p,r}=(\left\lfloor u_{r,N+\delta_{t}}\times N_{0}\right\rfloor \;mod\;K_{0})\times (v\left(Q_{t-1}\right)+1)$$

In cases where t mod 6=3 or t mod 6=4, the first integer that is larger than $I_{p,g}$ and coprime with 16 is set to be the integer key $J_{g,0}$ for the mutation operation on $T_{b,t-1}$. $H_{p,g}$ is computed with Equation (53), where $\gamma_{t}$ is defined as shown in Equation (43).

(53) $$H_{p,g}=(\left\lfloor u_{g,N+\gamma_{t}}\times N_{0}\right\rfloor \;mod\;K_{0})\times (v\left(Q_{t-1}\right)+1)$$

In cases where t mod 6=5 or t mod 6=0, the integer key $J_{b,0}$ for the mutation operation on $T_{b,t-1}$ is chosen to be the first integer that is larger than $H_{p,b}$ and coprime with 16. $H_{p,b}$ is computed with Equation (54), where $\varepsilon_{t}$ is defined by Equation (48).

(54) $$H_{p,b}=(\left\lfloor u_{b,N+\varepsilon_{t}}\times N_{0}\right\rfloor \;mod\;K_{0})\times (v\left(Q_{t-1}\right)+1)$$

$C_{b,t}$ is obtained by applying the mutation operation to $T_{b,t-1}$.

#### 2.3.3. The Encoding of Subsequences

Starting with t=1, $C_{b,t}$ is constructed from $C_{b,t-1}$ by applying the cross-over operation in Section 2.3.1 and the mutation operation in Section 2.3.2 to $C_{b,t-1}$. $C_{b,t}$ is queried to obtain the encoded subsequence $E_{t}$ for $Q_{t}$. $E_{t}$ can be obtained from $C_{b,t}$ and $Q_{t}$ by Equation (55).

(55) $$E_{t}=C_{b,t}(v\left(Q_{t}\right)+1)$$

After $E_{t}$ has been obtained, the value of t is incremented by 1. The above operations are repeated for the new t. A cipher image $C_{r}$ is obtained for P when all subsequences in $E_{p}$ have been encoded.

### 2.4. The Decryption Process

A cipher image $C_{r}$ can be decrypted by reversing the encryption operations applied to its plain image in a reversed order. Each pixel in $C_{r}$ is first decoded to generate a partially encoded image $E_{p}$. A set of sweeping operations is then applied to $E_{p}$ to obtain its corresponding scrambled image D. Finally, the bits in D are relocated to reverse the scrambling operations during encryption to obtain the plain image P.

#### 2.4.1. The Decoding of Pixels

The decoding of pixels in $C_{r}$ needs the values of $x_{r,0}$, $y_{r,0}$, $z_{r,0}$, $w_{r,0}$, $x_{g,0}$, $y_{g,0}$, $z_{g,0}$, $w_{g,0}$, $x_{b,0}$, $y_{b,0}$, $z_{b,0}$, $w_{b,0}$ utilized to implement the cross-over operations on a codebook and the values of $u_{r,0}$, ${\;u}_{g,0}$, and $u_{b,0}$ used to mutate a codebook during encryption.

The bits in the pixel values of $C_{r}\;$ are sequentially divided into subsequences of length 4. Specifically, for integers i and j such that 1≤i≤h and 1≤j≤w hold, an array $G_{r(i,j)}\;$ of 24 bits can be generated by merging the binary representations of the R, G, and B component values for the pixel in column j and row i of $C_{r}$, where $r\left(i,j\right)=\left(i-1\right)w+j$. Six subsequences $E_{6r\left(i,j\right)-5}$, $E_{6r\left(i,j\right)-4}$, $E_{6r\left(i,j\right)-3}$, $E_{6r\left(i,j\right)-2}$, $E_{6r\left(i,j\right)-1}$, and $E_{6r\left(i,j\right)}$ can be obtained by sequentially dividing the bits in $G_{r(i,j)}$ into regions of 4 bits. The decoding process starts with subsequence $E_{1}$ and sequentially decodes the other subsequences. For each positive integer t such that 1≤t<6hw holds, the decoding of $E_{t+1}$ is performed after the decoding of $E_{t}$ is complete.

Let $C_{b,0}$ be a codebook where the encoded subsequence of each subsequence is the subsequence itself. Starting with t=1, $C_{b,t}$ can be obtained by processing $C_{b,t-1}$ by the cross-over operation in Section 2.3.1 and the mutation operation in Section 2.3.2. $C_{b,t}$ is queried to obtain the decoded subsequence $Q_{t}$ for $E_{t}$. $Q_{t}$ can be determined by searching $C_{b,t}$ to find the location of the entry that matches $E_{t}$. After $Q_{t}$ has been determined, increment the value of t by 1 and repeat the above operations for the new t. The partially decrypted image $E_{p}$ is obtained from $C_{r}$ when the decoding of all subsequences in $C_{r}$ has been performed.

#### 2.4.2. Reversing the Sweeping Operations

The scrambled image D that corresponds to $E_{p}$ can be obtained by reversing the accumulative bit exclusive-or operations utilized to generate $\;E_{p}$ from D. An array of 24 bits can be constructed by merging the binary representations of the R, G, and B component values for each pixel in $E_{p}$. Let $U_{r(i,j)}$ denote the bit array for the pixel in column j and row i of $E_{p}$, where $r\left(i,j\right)=\left(i-1\right)h+j$, the bits in each column of $E_{p}$ are processed by a bottom-up accumulative bit exclusive-or operation. For column $j_{c}$ in $E_{p}$, where $1\leq j_{c}\leq w$ holds, the bit array of each pixel in the column is processed by a sweeping operation as described by Equations (56) and (57), where k is a positive integer such that 1<k≤h holds.

(56) $$M_{c,h}=SW(0,U_{\left(h-1\right)w+j_{c}})$$

(57) $$M_{c,k-1}=SW(M_{c,k}\left(24\right),\;U_{(k-2)w+j_{c}})$$

For each k, the bits in $M_{c,k}$ are assigned to $U_{\left(k-1\right)w+j_{c}}$ after $M_{c,1},M_{c,2},\ldots M_{c,h}$ are determined.

A top-down accumulative bit exclusive-or operation is applied to the bit arrays of all pixels in each column of $E_{p}$. For column $j_{c}$ in $E_{p}$, the bit array of each pixel in the column is processed by a sweeping operation as described by Equations (58) and (59), where k is an integer that satisfies 1<k≤h.

(58) $$N_{c,1}=SW(0,U_{j_{c}})$$

(59) $$N_{c,k}=SW(N_{c,k-1}\left(24\right),\;U_{\left(k-1\right)w+j_{c}})$$

For each k, $U_{\left(k-1\right)w+j_{c}}$ contains the bits in $N_{c,k}$ after $N_{c,1},N_{c,2},\ldots N_{c,h}$ are determined.

After the sweeping operations on all columns in $E_{p}$ have been reversed, a right-to-left accumulative bit exclusive-or operation is used to process the bit arrays of all pixels in each row of $E_{p}$. For row $i_{r}$ in $E_{p}$, where $i_{r}$ is an integer that satisfies $1\leq i_{r}\leq h$, the bit array of each pixel in the row is processed by a sweeping operation as shown in Equations (60) and (61), where $b_{r}=\left(i_{r}-1\right)w$ and k is a positive integer such that 1<k≤w holds.

(60) $$P_{l,w}=SW(0,U_{b_{r}+w})$$

(61) $$P_{l,k-1}=SW(P_{l,k}\left(24\right),\;U_{b_{r}+k-1})$$

The bits in $U_{b_{r}+k}$ are assigned to be those in $P_{l,k}$ for each k after $P_{l,1},\;P_{l,2},\ldots P_{l,w}$ have been determined.

Finally, a left-to-right accumulative bit exclusive-or operation is applied to the bit arrays of all pixels in each row of $E_{p}$. For row $i_{r}$ in $E_{p}$, a sweeping operation described by Equations (62) and (63) is utilized to process the bit array of each pixel in the row. Similarly, k is a positive integer such that 1<k≤w holds.

(62) $$Q_{l,1}=SW(0,U_{b_{r}+1})$$

(63) $$Q_{l,k}=SW(Q_{l,k-1}\left(24\right),\;U_{b_{r}+k})$$

After $Q_{l,1},\;Q_{l,2},\ldots Q_{l,w}$ are determined, $U_{b_{r}+k}$ are set to contain the bits in $Q_{l,k}$ for each k. The scrambled image D is recovered from $E_{p}$ when all sweeping operations that convert D into $E_{p}$ are reversed.

#### 2.4.3. Recovery of a Plain Color Image

The operations for scrambling can be reversed to recover the plain image P of $C_{r}$ from D. Specifically, the binary bits that represent the values of pixels in D are partitioned into 24 sequences $L_{1},L_{2},\ldots ,L_{24}$ with the approach in Section 2.1. Two sequences of real numbers $y_{1},y_{2},\ldots ,y_{N},\ldots$ and $z_{1},z_{2},\ldots ,z_{N},\ldots$ are generated based on the initial values $y_{0}$ and $z_{0}$ used in the scrambling process. For each pair of integers (i,j) that satisfy 1≤i≤h and 1≤j≤w, the mapping $\sigma_{r(i,j)}$ in Equation (4) is constructed for the pixel that is in column j and row i of P.

Based on the mappings for all pixels in D, the partition of the bits in  D can be performed using the mappings recursively defined in Equations (6) and (7). For each k that satisfies 1≤k≤24, a sequence $L_{k}$ of hw bits is generated by setting the bit in position t of $L_{k}$ to be $U_{t}(\mu_{t}\left(k\right))$ for each integer t such that 1≤t≤hw holds. The bits in $\;L_{k}$ need to be relocated to their original positions in the plain image  P to recover P. Specifically, an array $A_{c}$ that consists of hw integers are constructed. For each integer $i_{l}$ such that $1\leq i_{l}\leq hw$ holds, $A_{c}(i_{l})$ is set to be $i_{l}$. The scrambling operation described in Section 2.1 on $S_{k}$ is performed on $A_{c}$. The resulting array $A_{s}$ thus contains the original position of each bit in $L_{k}$. A sequence $S_{k}$ can be obtained by relocating the bits in $L_{k}$ to their original positions by applying the operation defined in Equation (64) for each integer $i_{l}$ such that $1\leq i_{l}\leq hw$ holds.

(64) $$S_{k}\left(A_{s}\left(i_{l}\right)\right)=L_{k}(i_{l})$$

The plain image P can be constructed from the recovered sequences $S_{1},S_{2},\ldots ,S_{24}$. Specifically, $S_{k}(t)$ is assigned to the position $\mu_{t}\left(k\right)$ of $U_{t}$ for each pair of integers (k,t) that satisfies 1≤k≤24 and 1≤t≤hw. As a result, the plain image P for $C_{r}$ can be obtained.

### 2.5. Computational Complexity

Given a color image that contains w columns and h rows, the binary bits in such an image can be partitioned into 24 sequences of bits in O(hw) time. It is also clear that O(hw) time is needed to relocate the bits in each sequence. A scrambled image can thus be obtained in O(hw) time.

The sweeping operations defined in Equations (21) and (22) require O(w) time to process the pixels in one row of a scrambled image. Similarly, the sweeping operations defined in Equations (23) and (24) need O(w) time to process one row. The sweeping operations defined by Equations (25) and (26) can be accomplished in O(h) time. In addition, O(h) time is needed to complete the bit exclusive-or operations described in Equations (27) and (28). The sweeping process can thus be accomplished in O(hw) time.

The encoding of a pixel in a partially encrypted image requires the construction of a codebook based on the information generated by a number of chaotic systems. Since both the cross-over and mutation operations can be performed in O(1) time, the codebook for the encryption of a single subsequence can thus be constructed in O(1) time. Therefore, the encoding of a subsequence can be accomplished in O(1) time. The encoding of all subsequences in a partially encrypted image needs O(hw) time. The total amount of computation time needed to encrypt a plain color image is thus O(hw).

A cipher image with h rows and w columns is decrypted by reversing the operations in the encryption process. It is clear that all operations in the encryption can be reversed in O(hw) time. The plain image of a cipher image thus can be obtained in O(hw) time.

## 3. Results

An implementation of this encryption approach has been generated based on MATLAB. Seven benchmark images and a number of color images in the BSD dataset [40] have been utilized to test and evaluate its performance. Cipher images provided by this encryption approach are evaluated using a few measures and its performance is compared with that of several state-of-the-art encryption methods [3,22,27,28,35,42,43,44,45,46,47,48] based on these measures. The values of parameters $\;N,{\;N}_{0}$, ${\;K}_{0}$, and $\tau_{0}$ are set to be 100, $10^{8}$, 1000, and 2, respectively, for all the experiments.

### 3.1. Attacks That Utilize Brutal Forces

Attacks that utilize brutal forces generally exhaustively search within the key space for encryption and determine the combination of keys that can recover the plain image from a cipher image. The key space size of an approach is thus a measure of its ability to protect cipher images from attacks that utilize brutal force. The proposed approach partitions the binary bits in a plain image into 24 subsequences for scrambling. A rectangular structure is constructed to relocate the bits in each subsequence. For a rectangular structure with $r_{h}$ rows and $c_{w}$ columns, the relocation performed on the rectangular structure requires $r_{h}+c_{w}$ integers to move the bits in the rectangular structure to new locations. In practice, integers for scrambling 20 of the 24 subsequences are considered parameters that can be optimized for improved encryption security. The integers needed to scramble the remaining 4 subsequences are used as encryption keys. Let $M_{1}$ be the number of integers that an integer key associated with a column or row can be chosen from, ${M_{1}}^{{4(r}_{h}+c_{w})}$ different combinations of integer keys for the rows and columns in a rectangular structure thus need to be considered in an exhaustive search. The encoding process requires the outputs of 15 different chaotic systems to change the codebook for encoding. Let $M_{2}$ be the size of the set of numbers that can be chosen as the initial value for the chaotic system, an exhaustive search needs to consider ${M_{2}}^{15}$ different combinations. The key space of the proposed approach thus has a lower bound of ${M_{1}}^{4(r_{h}+c_{w})}{M_{2}}^{15}$ for its size.

In the case where a color image that needs to be encrypted contains at least $10^{4}$ pixels, a lower bound of $2\sqrt{r_{h}r_{w}}\geq 200$ exists for the value of $r_{h}+r_{w}$. For practical applications, $M_{1}\geq 2$ and $M_{2}\geq 2^{50}$, the key space of the proposed approach thus has a lower bound of $2^{850}$ for its size. The key space size of the proposed encryption approach is compared with those of several other state-of-the-art encryption methods. Table 1 shows the comparison. Table 1 clearly shows that the key space of the proposed approach is larger than most other encryption methods in size. It is thus more secure than most other methods under attacks of brutal force.

In cases where a key is represented by 32 binary bits, Equation (30) suggests that the number of keys that can generate a given value for $x_{k}$ is not larger than 32. In addition, it is clear from Equations (34)–(54) that at most $\left\lceil N_{0}/K_{0}\right\rceil$ different values of $x_{k}$ can lead to the same integer key for encoding. In practice, $N_{0}$ and $K_{0}$ can be selected such that $\left\lceil N_{0}/K_{0}\right\rceil \leq 2^{20}$ holds. Since 15 different chaotic systems are altogether utilized for the encoding of a partially encrypted image, the total number of combinations of keys that are equivalent is at most ${\left(32\times 2^{20}\right)}^{15}=2^{375}$. Since the size of the key space is at least $2^{850}$, at least $2^{475}\;$ combinations of keys are mutually not equivalent.

### 3.2. Statistical Attacks

**Figure 4:**
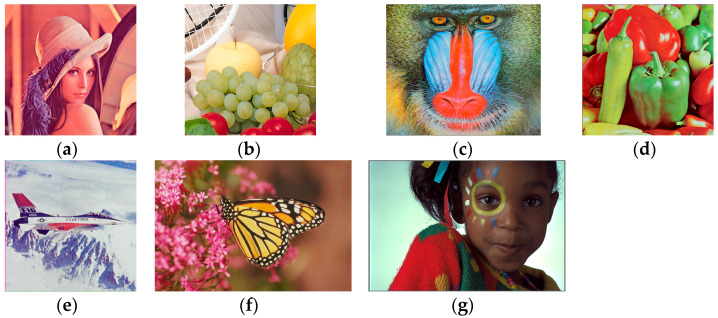
Benchmark color images utilized to test the proposed approach. (**a**) image Lena; (**b**) image Fruits; (**c**) image Baboon; (**d**) image Peppers; (**e**) image Airplane; (**f**) image Monarch; (**g**) image Girl.

As part of the experiments, the proposed approach is utilized to encrypt seven benchmark color images and the cipher images obtained on them are analyzed to evaluate their security under statistical attacks. Figure 4a–g show the seven color benchmark images. Two of these images, with the names Girl and Monarch, are 768 × 512 images. The remaining five images, with names Lena, Fruits, Baboon, Peppers, and Airplane, are 512 × 512 images.

#### 3.2.1. Histogram Analysis of Cipher Images

**Figure 5:**
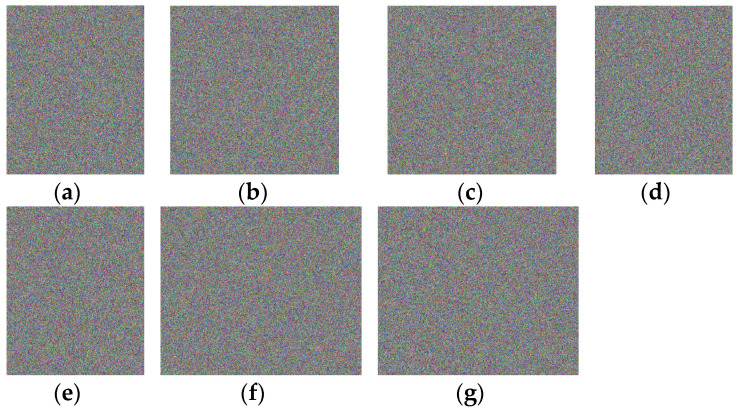
Cipher images obtained by the proposed encryption method. (**a**–**g**) are the results of encryption for Lena, Fruits, Baboon, Peppers, Airplane, Monarch, and Girl, respectively.

Figure 5a–g show the cipher images generated on all benchmark images with the proposed approach. It is clear from Figure 5 that the cipher images contain randomized data and the original contents in the benchmark images are invisible in their cipher images.

**Figure 6:**
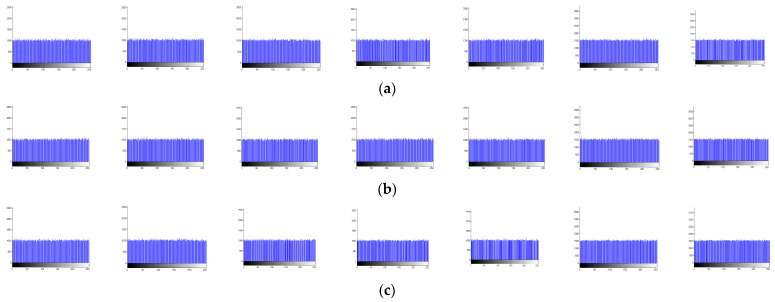
Histograms obtained from the values of R, G, and B components in the cipher images. (**a**–**c**), respectively, show the histograms for the values of R, G, and B components; in each row, histograms for the cipher images of Lena, Fruits, Baboon, Peppers, Airplane, Monarch, and Girl are shown in order from left to right.

The histograms of the cipher images for R, G, and B component values are shown in Figure 6a–c). Figure 6 clearly shows that the distributions of the values of R, G, and B components in all cipher images are close to a uniform distribution. The image contents in the benchmark images cannot be obtained by analyzing the histograms of their cipher images.

The variance of histograms is a measure often utilized to compare the distribution described in a histogram with a uniform distribution [31]. Let $H_{c}$ be a histogram that provides a distribution over integers from 0 to 255. The variance of histogram $Var(H_{c})$ of $H_{c}$ is defined as shown in Equation (35).

(65) $$Var\left(H_{c}\right)=\frac{1}{2m^{2}}\sum_{i=1}^{m}\sum_{j=1}^{m}{\left(h_{i}-h_{j}\right)}^{2}$$

where m=256 is the number of integers that can be the value of an R, G, or B component in a pixel. $h_{i}$ is the integer associated with value i in $H_{c}$ and $h_{j}$ is the integer associated with value j in $H_{c}$. Equation (65) clearly shows that a lower variance of histograms indicates that the corresponding histogram is closer to a distribution that is uniform.

Table 2 shows the variances of histograms for the cipher images provided by the proposed approach and the encryption methods developed in [20,28,31,47,48]. Table 2 suggests that the performance of the proposed approach on Lena and Airplane is the highest of all tested methods. In addition, its performance is the second best on Fruits, Baboon, Peppers, and Monarch. The overall performance of the proposed method on the variance of histograms is thus better than that of the other tested approaches.

#### 3.2.2. Analysis of Correlations

**Figure 7:**
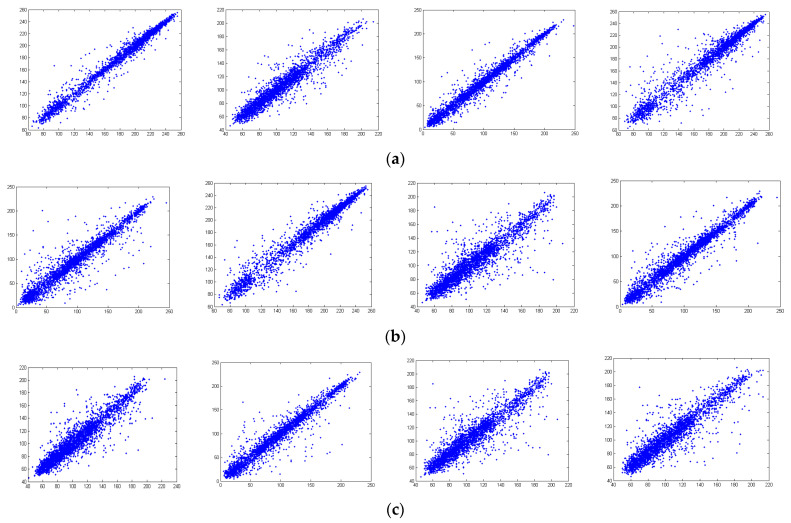
Correlations in different directions in Lena for the component values of R, G, and B. (**a**–**c**), respectively, are the correlation plots for R, G, and B components. In each row, correlations obtained for the horizontal, vertical, diagonal, and anti-diagonal directions are shown from left to right.

**Figure 8:**
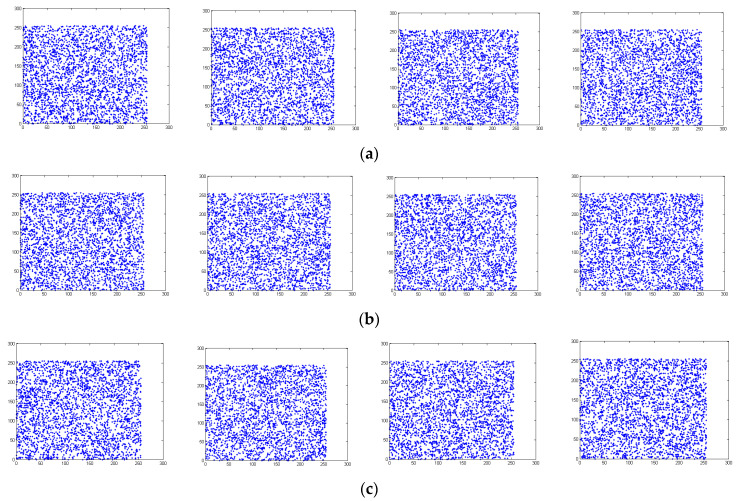
Correlations in four different directions for the component values of R, G, and B in the cipher image of Lena. (**a**–**c**) are the correlation plots for the component values of R, G, and B, respectively. Correlations in the horizontal, vertical, diagonal, and anti-diagonal directions are shown from left to right in each row.

Pixels that are close in a plain image are often strongly correlated in their pixel values. Ideally, the correlations that exist locally among such pixels in a cipher image must be significantly reduced to ensure its strength against statistical attacks. In practice, the local correlations among pixels in a cipher image cannot be removed completely and their values are thus important measures for the security of an encryption result. Figure 7a–c show the correlations of neighboring pixels in four different directions in Lena for the R, G, and B components. The four directions include the horizontal, vertical, diagonal, and anti-diagonal directions. Figure 8a–c show the correlations of pixels that are adjacent in these four directions for the component values of R, G, and B. The plots in both figures are generated with 3000 pixels chosen in the corresponding image at random. Figure 7 and Figure 8 suggest that the proposed encryption method can significantly reduce the correlations that often exist locally in a plain image.

Table 3 shows the local correlations between neighboring pixels in the benchmark images and their encryption results. It is clear from Table 3 that, for all benchmark images, the correlations in the four directions have been reduced to values near zero after encryption. The encryption results provided by the proposed method are thus highly secure under statistical attacks. Table 4 compares the correlations for R components in the encryption results generated by all tested approaches on Lena. Table 4 clearly demonstrates that the proposed encryption method achieves the lowest correlation in a vertical direction and its performance on correlations in other directions is comparable with the best performance that can be achieved by the other tested approaches.

### 3.3. Differential Attacks

**Figure 9:**
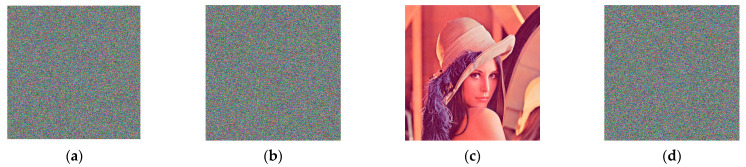
A comparison of cipher images and decrypted images obtained on Lena with one of the keys slightly perturbed; (**a**) the cipher image obtained with the original values of keys; (**b**) the cipher image obtained with the perturbed values of keys; (**c**) the decrypted image obtained from the cipher image in (**a**) with the original values of keys; (**d**) the decrypted image obtained from the cipher image in (**b**) with the original values of keys.

A tiny amount of change is often introduced to the keys for encryption or the image that needs to be encrypted to test the strength of an encryption approach against differential attacks. Specifically, one of the initial values of the chaotic systems for encoding is changed by a small amount of $10^{-16}$ and the other keys remain the same, a new cipher image can be obtained by encrypting Lena based on the new values of keys. Figure 9a shows the cipher image of Lena obtained with the original values of keys and Figure 9b is the cipher image of Lena generated based on the new values of keys. Figure 9c is the decrypted image obtained from the cipher image in Figure 9a with the original key values. Figure 9d is the decryption result generated from the cipher image in Figure 9b with the new key values. Figure 9 clearly shows that encryption results obtained with the proposed approach are highly sensitive to key values and a slightly different set of key values cannot lead to the corresponding plain images by decryption.

**Figure 10:**
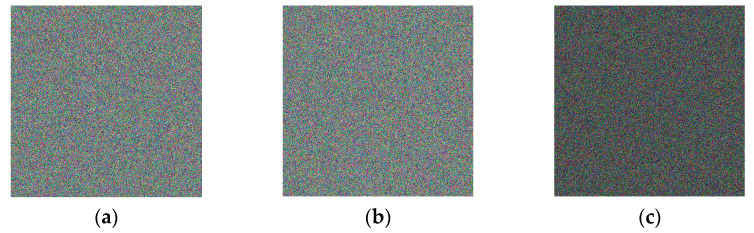
A comparison of cipher images obtained on Lena and a perturbed plain image of Lena; (**a**) the cipher image obtained on Lena; (**b**) the cipher image obtained on the perturbed Lena; (**c**) the difference between the cipher images in (**a**,**b**).

Similarly, a component in a pixel of Lena is changed by 1 to obtain a plain image slightly different from Lena. The new plain image is encrypted with the same set of key values. Figure 10a is the cipher image of Lena and Figure 10b shows the cipher image of the perturbed image of Lena. The difference image of the two cipher images in Figure 10a,b is shown in Figure 10c. It is clear from Figure 10 that cipher images generated based on the proposed approach are highly sensitive to plain images and the cipher image of a plain image cannot be obtained from that of a similar plain image.

**Figure 11:**
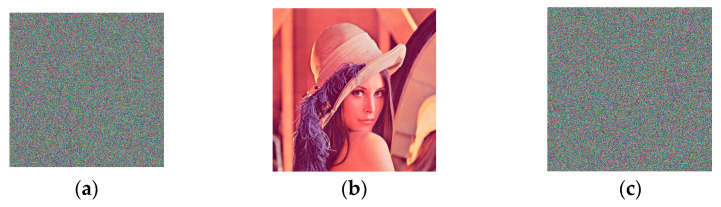
Decrypted images of a cipher image of Lena and its perturbed cipher image; (**a**) a cipher image of Lena; (**b**) the decrypted image of the cipher image in (**a**); (**c**) the decrypted image of the perturbed cipher image of Lena.

Figure 11 shows the decrypted images of two highly similar cipher images. A component in a pixel of a cipher image of Lena is changed by 1 and the resulting image is a perturbed cipher image of Lena. Both images are decrypted with the key values used to generate the cipher image. Figure 11a is the cipher image of Lena and Figure 11b shows the decrypted image of the image in Figure 11a. Figure 11c is the decrypted result obtained on the perturbed cipher image. Figure 11 suggests that the plain image of a cipher image cannot be inferred from that of a similar cipher image.

Two sensitivity measures, including the number of pixels change rate (NPCR) and unified average changing intensity (UACI), are generally used to test the sensitivity of an encryption approach to tiny amounts of perturbation in the keys of encryption or plain image [20,31,35,47]. Specifically, let P be a plain color image that contains w columns and h rows, and let C be the cipher image generated based on a given set of values for encryption keys. After one of the encryption keys or a pixel component in the plain color image is changed by a tiny amount, the plain image is encrypted with the same encryption method and a new cipher image $C_{1}$ is generated. For a component m, Equations (66) and (67) can be utilized to compute its NPCR and UACI values, where the value of F(u,v,m) is 0 if C(u,v,m) and $C_{1}\left(u,v,m\right)$ are equal and is 1 otherwise.

(66) $$N\left(m\right)=\frac{\sum_{u=1}^{h}\sum_{v=1}^{w}F(u,v,m)}{hw}$$

(67) $$U\left(m\right)=\frac{\sum_{u=1}^{h}\sum_{v=1}^{w}\left|C_{1}\left(u,v,m\right)-C\left(u,v,m\right)\right|}{255hw}$$

For two independent random color images, $C_{1}$ and C, a value of 99.6094 is obtained for NPCR and a value of 33.4635 is obtained for UACI.

The initial value for a chaotic system that performs encoding is changed by an amount of $10^{-16}$ to test the key sensitivity of this encryption approach. For each benchmark image, Equations (66) and (67) are utilized to determine the NPCR and UACI for each component. The NPCR and UACI values for key sensitivity can be found in Table 5. Table 5 clearly demonstrates that the key sensitivity values for the benchmark images in all components are near the values that can be obtained on two completely independent random images.

For NPCR randomness tests, the work in [49] suggests that lower bound values of 99.5893, 99.5810, and 99.5717 are used for testing levels of 0.001, 0.01, and 0.05, respectively. From Table 5, it is clear that this encryption method is able to pass the test of NPCR randomness at all three levels for each benchmark image. For the tests of UACI randomness [49], lower bound values of 33.3730, 33.3445, and 33.3115 are used for testing levels of 0.001, 0.01, and 0.05, respectively, while the upper bound values of 33.5541, 33.5826, and 33.6156 are used for the three testing levels. The results in Table 5 clearly indicate that this encryption approach is able to pass the test of UACI randomness at all three levels for each benchmark image.

Table 6 compares the key sensitivity of the proposed encryption method with several other encryption methods in both UACI and NPCR values obtained on Lena. The results in Table 6 show that a mean value of 99.6100 is achieved by the proposed approach on NPCR values for key sensitivity and the mean value of UACI for the proposed approach is 33.4733. Due to the fact that the best values for NPCR and UACI are 99.6064 and 33.4635, respectively, this encryption method can thus achieve performance that is near optimal and comparable with the best performance achieved by other methods on key sensitivity.

With this encryption approach on plain image sensitivity, a pixel is randomly selected in a plain image and one of its pixel components is changed by 1 and the resulting image is encrypted by the proposed approach. Equations (66) and (67) are employed to calculate the UACI and NPCR values for each benchmark image. Table 7 shows the NPCR and UACI values obtained on each benchmark image for plain image sensitivity. It is clear that all plain image sensitivity values in Table 7 are close to the values obtained on two completely independent random images.

Table 7 suggests that this encryption approach can pass the randomness tests at all three levels for plain image sensitivity in terms of both UACI and NPCR values. A comparison of the plain image sensitivity values for all tested encryption methods on Lena can be found in Table 8. Table 8 clearly shows that the proposed encryption method can achieve a mean value of 99.6100 on NPCR values for plain image sensitivity. The mean of the plain image sensitivity UACI values for the proposed encryption method is 33.4933. The proposed encryption method can thus achieve near-optimal performance on plain image sensitivity. In addition, its performance is comparable with that of other encryption methods on plain image sensitivity.

### 3.4. Information Entropies of Encryption Results

In general, the randomness associated with an encryption result can be effectively measured using its information entropies. The information entropy of a cipher image in all three components can be calculated from its histograms $H_{c}$ based on Equations (68) and (69), where m=1,2,3 is an integer that represents the R, G, and B components of the cipher image, respectively, w and h are the numbers of columns and rows in the image, and $H_{c}(s,m)$ is the number of pixels that have a pixel value of s for component m.

(68) $$p\left(s,C,m\right)=\frac{H_{c}(s,m)}{hw}$$

(69) $$E\left(C,t\right)=-\sum_{s=0}^{255}p\left(s,C,m\right)\log_{2}p(s,C,m)$$

Equation (69) suggests that a uniform distribution over all integers between 0 and 255 has a value of 8.0 for its information entropy, the best information entropy an encrypted image can achieve for a pixel component is thus 8.0.

Table 9 shows the information entropies of all three components in the encryption results obtained with the proposed encryption method. Table 9 demonstrates that the proposed encryption method is able to achieve near-optimal information entropies for all components in cipher images.

Table 10 compares the information entropies of the encryption results provided by all encryption methods for Lena. It is clear from Table 10 that the proposed encryption method outperforms all other tested methods on the information entropies of components G and R. Its performance on component B is comparable with the best performance that can be achieved by the other tested encryption methods.

### 3.5. Values of Peak Noise Signal Ratio

The amount of difference between a color image and its encryption result can be represented by the peak signal noise ratio (PSNR). Given a color image P that contains h rows and w columns, let C be its encryption result. The PSNR of C can be obtained with Equations (70) and (71). Cipher images with a higher PSNR value are thus usually more secure [20].

(70) $$MSE\left(P,C\right)=\sum_{u=1}^{h}\sum_{v=1}^{w}\sum_{m=1}^{3}{\left|P\left(u,v,m\right)-C\left(u,v,m\right)\right|}^{2}$$

(71) $$PSNR\left(C,P\right)=20\log_{10}\frac{255\sqrt{3hw}}{\sqrt{MSE(P,C)}}$$

Table 11 provides the PSNR values for the encryption results obtained with all tested encryption methods. It can be seen from Table 11 that the performance of the proposed method is comparable with that of the approaches in [27,28]. In addition, it outperforms most of the other tested encryption approaches on PSNR values for cipher images.

### 3.6. Encryption of General Color Images

For a more comprehensive evaluation of its performance, 100 color images in the BSD500 dataset [40] are encrypted by the proposed approach. The size of all color images used in the experiment is 481 × 321. In addition, the performance of the methods proposed in [20,27,28,31,35], is evaluated and compared with that of the proposed encryption method using the same set of images.

For the variances of histograms, Table 12 provides their means and standard deviations for the encryption results obtained with all tested methods. For the variances of histograms, the results in Table 12 clearly show that the proposed encryption method can achieve performance higher than that of the approaches in [20,31,35]. Its performance is comparable with the best performance that can be achieved by the approaches in [27,28] on the variances of histograms.

A comparison of the information entropies of encryption results provided by all tested encryption methods can be found in Table 13. Table 13 demonstrates that the proposed encryption method is able to achieve a mean value of 7.9988 for the information entropies of all encryption results. Its overall performance on information entropies is thus comparable with the best performance of the approach in [28] and is slightly better than the best performance of the approaches in [20,27,31,35]. Table 14 provides statistical information on the PSNR values of the encryption results generated with all tested methods. Table 14 clearly shows that the proposed encryption method can achieve a performance comparable with the best performance of all other tested approaches on PSNR values.

The overall performance of several encryption methods on key sensitivity is compared in Table 15. Table 15 clearly demonstrates that the means of both UACI and NPCR values are near their best values for all components and the overall performance of the proposed encryption method on key sensitivity is better than the overall performance of other methods. Table 16 provides a comparison of the overall performance on plain image sensitivity for all tested methods. Table 16 indicates that the overall performance of the proposed encryption method is comparable with that of the other methods on plain image sensitivity.

### 3.7. Analysis of Classical Attacks

Four different categories of classical attacks have been identified and summarized in previous work [50], the major features of these attacks can be described as follows.

1. Known plaintext attacks. In these attacks, a certain amount of plaintext and its ciphertext are available and can be utilized during attacks.
2. Chosen ciphertext attacks. In such attacks, a computer program that implements the decryption algorithm can be accessed by an attacker to decrypt some selected special ciphertext and its plaintext can be obtained.
3. Chosen plaintext attacks. In attacks of this type, a computer program that implements the encryption algorithm can be utilized by an attacker to encrypt some special plaintext and its ciphertext can be generated.
4. Ciphertext-only attacks, in such types of attacks, a certain amount of ciphertext is available to an attacker.

It is clear from the above descriptions of the four classical attacks that chosen ciphertext attacks and chosen plaintext attacks are more powerful than the other types of attacks. An encryption approach can properly handle all four types of classical attacks if it is capable of defending its cipher images against the chosen ciphertext attacks and chosen plaintext attacks. In these two different types of attacks, an attacker obtains equivalent encryption keys by analyzing the plaintext of some special ciphertext or the ciphertext of some special plaintext. Section 2 clearly shows that the equivalent encryption keys of the proposed approach are dependent on the contents of a plain image. The equivalent keys obtained by an attacker thus cannot lead to the plain image of a cipher image since a different set of equivalent keys is needed to decipher the cipher image. Cipher images provided by the proposed approach are thus secure under both chosen ciphertext attacks and chosen plaintext attacks. The proposed approach is thus able to properly handle classical attacks from all four categories.

## 4. Discussion

The proposed encryption method has been evaluated based on several benchmark color images and a set of images from the BSD dataset. However, its overall performance on all possible types of plain color images remains uncertain and thus needs to be evaluated by experiments that are more comprehensive. The encoding operations in the proposed encryption method are based on a codebook that is under constant evolution and updates determined by a set of chaotic systems. It is expected that encodings performed on longer subsequences can possibly further improve the randomness of an encryption result and thus lead to improved security for a cipher image. However, for a subsequence of l bits, a codebook with $2^{l}$ entries needs to be constructed and maintained for encryption. Such an exponential growth in the size of a codebook requires that l must be a small integer. The exponential size of the codebook used for encoding is thus an important disadvantage of the proposed approach. Further improvements in this aspect may need to be considered in future work.

In [51], an encryption method that combines bit-level scrambling with multiplication diffusion is proposed for image encryption. Similar to the proposed approach, the bit-level scrambling operations developed in [51] partitions the bits in an image into eight sets for scrambling. However, the method in [51] scrambles bits based on a combination of flip scrambling, improved circle index scrambling, and binary tree while the bit-level scrambling method proposed in this paper utilizes a rectangular structure and a set of logistic systems to scramble the bits. The techniques developed in [51] for bit-level scrambling can potentially be used together with the proposed approach to further improve the security of cipher images.

## 5. Conclusions

A new method is proposed in this paper to generate secure encryption results for color images. A color image can be encrypted in three phases. In the first phase, the proposed approach partitions the binary bits in the pixel values of an image into a few sequences and scrambles the bits in each sequence to obtain a fully scrambled color image. In the second phase of the approach, a scrambled image is processed with a set of accumulative bit exclusive-or operations performed on all columns and rows the image contains. The resulting image is an image that is partially encrypted. The third phase of the approach utilizes an evolutionary codebook to encode the partially encrypted image and generate the encryption result. The codebook is constantly updated with the cross-over and mutation operations determined by the outputs of a number of chaotic systems. Analysis of the proposed encryption method on its key space demonstrates that it is secure under attacks that utilize brutal forces. Testing results show that the encryption results provided by the proposed method are secure under different types of attacks. Its performance is also compared with that of several other encryption methods based on several different measures for security. The results of the comparison suggest that it is able to achieve overall performance higher than that of several other approaches developed to encrypt images. The proposed approach can thus be used in various applications that require the secure encryption of color images.

For future research, more comprehensive analysis and experiments are needed to evaluate the overall performance of the proposed approach. In addition, more sophisticated cross-over and mutation operations can be developed to further improve the security of encryption. The work in [52] analyzes the bit-level scrambling operations used in an encryption approach based on chaotic systems and develops a chosen ciphertext attack method that can crack the approach. It is, therefore, necessary to consider the bit-level operations developed in other research work [51,52] to further improve the randomness of scrambled images.

## Figures and Tables

**Table 1:** A comparison of the proposed approach and a few other methods for image encryption on key space sizes.

Encryption Approaches	Key Space Size
The proposed	$2^{850}$
Ref. [28]	$2^{3320}$
Ref. [27]	$2^{2092}$
Ref. [22]	$2^{199}$
Ref. [35]	$2^{170}$
Ref. [43]	$2^{241}$
Ref. [3]	$2^{104}$
Ref. [44]	$2^{128}$
Ref. [45]	$2^{128}$
Ref. [46]	$2^{128}$

**Table 2:** The variance of histograms for cipher images generated with all tested methods for image encryption.

Images	The Proposed	Ref. [28]	Ref. [31]	Ref. [20]	Ref. [48]	Ref. [47]
Lena	984.56	1040.39	1054.78	1047.40	1027.59	1043.21
Fruits	996.16	951.94	1034.23	1026.51	1082.34	1013.32
Baboon	953.27	1024.29	901.22	1098.64	1058.13	1061.04
Peppers	970.79	1146.23	1037.78	1046.25	946.67	1105.72
Airplane	957.15	1082.83	1268.02	1132.27	1103.16	1141.38
Monarch	1537.37	1409.30	1607.51	1564.39	1563.72	1653.27
Girl	1533.70	1536.50	1543.23	1513.42	1530.21	1620.12

**Table 3:** The correlations between pixels that are adjacent in four directions in the testing benchmark images and their encryption results provided by the proposed approach.

Images	Directions	Plain Image			Cipher Image		
		R	G	B	R	G	B
Lena	V	0.9753	0.9666	0.9334	−0.0003	0.0017	−0.0007
	H	0.9853	0.9802	0.9558	−0.0026	−0.0003	−0.0006
	D	0.9734	0.9630	0.9264	0.0022	−0.0014	0.0002
	A	0.9648	0.9536	0.9198	−0.0007	0.0019	−0.0019
Fruits	V	0.9936	0.9855	0.9265	0.0027	−0.0010	−0.0020
	H	0.9928	0.9848	0.9192	−0.0001	−0.0007	0.0013
	D	0.9897	0.9783	0.8809	0.0013	0.0011	−0.0008
	A	0.9868	0.9694	0.8531	0.0002	0.0029	−0.0028
Baboon	V	0.9235	0.8668	0.9067	−0.0028	−0.0020	0.0010
	H	0.8740	0.7759	0.8844	0.0009	0.0025	−0.0004
	D	0.8649	0.7432	0.8544	0.0015	−0.0020	−0.0028
	A	0.8670	0.7494	0.8540	−0.0007	0.0025	0.0029
Peppers	V	0.9635	0.9811	0.9665	−0.0012	−0.0018	−0.0027
	H	0.9663	0.9817	0.9664	−0.0004	−0.0007	−0.0022
	D	0.9563	0.9686	0.9477	−0.0043	−0.0006	−0.0038
	A	0.9585	0.9708	0.9477	0.0008	0.0020	0.0015
Airplane	V	0.9726	0.9578	0.9640	−0.0003	−0.0008	0.0019
	H	0.9568	0.9678	0.9353	0.0001	−0.0007	0.0041
	D	0.9343	0.9326	0.9146	0.0018	0.0044	−0.0010
	A	0.9350	0.9300	0.9075	−0.0022	−0.0017	−0.0027
Monarch	V	0.9648	0.9523	0.9566	−0.0002	0.0015	0.0013
	H	0.9597	0.9453	0.9506	−0.0004	−0.0016	−0.0009
	D	0.9450	0.9252	0.9309	0.0028	−0.0003	0.0007
	A	0.9245	0.8984	0.9118	−0.0011	−0.0003	−0.0004
Girl	V	0.9811	0.9887	0.9866	0.0048	0.0021	0.0009
	H	0.9901	0.9937	0.9927	0.0032	−0.0008	−0.0002
	D	0.9750	0.9848	0.9823	0.0036	0.0021	−0.0012
	A	0.9772	0.9858	0.9832	0.0001	0.0008	−0.0005

**Table 4:** The correlations for neighboring pixels in three directions in the encryption results provided by the proposed encryption method and several other approaches for Lena.

Methods	H	V	D
The Proposed	−0.0026	−0.0003	0.0022
Ref. [28]	0.0028	0.0019	−0.0011
Ref. [35]	0.0046	−0.0028	0.0014
Ref. [31]	0.0012	0.0035	0.0056
Ref. [20]	0.0021	0.0018	−0.0026
Ref. [32]	−0.0026	−0.0038	0.0017
Ref. [27]	0.0023	−0.0020	0.0013

**Table 5:** Values of key sensitivities for the proposed encryption method in NPCR and UACI in all three components.

Images	NPCR(%)			UACI(%)		
	R	G	B	R	G	B
Lena	99.61	99.62	99.60	33.48	33.49	33.45
Fruits	99.60	99.61	99.61	33.47	33.52	33.38
Baboon	99.60	99.60	99.60	33.46	33.45	33.46
Peppers	99.61	99.61	99.63	33.44	33.49	33.47
Airplane	99.62	99.60	99.60	33.47	33.47	33.44
Monarch	99.60	99.63	99.61	33.47	33.47	33.45
Girl	99.60	99.64	99.61	33.43	33.52	33.43

**Table 6:** The NPCR and UACI values for the key sensitivity of all tested encryption methods on Lena.

Methods	NPCR(%)			UACI(%)		
	R	G	B	R	G	B
The Proposed	99.61	99.62	99.60	33.48	33.49	33.45
Ref. [28]	99.61	99.60	99.63	33.45	33.42	33.51
Ref. [20]	99.57	99.58	99.57	33.35	33.37	33.38
Ref. [27]	99.62	99.62	99.62	33.48	33.45	33.50
Ref. [31]	99.60	99.59	99.61	33.45	33.45	33.45
Ref. [35]	99.61	99.61	99.61	33.47	33.47	33.47

**Table 7:** Plain image sensitivities of the proposed encryption method in NPCR and UACI for all three components.

Images	NPCR(%)			UACI(%)		
	R	G	B	R	G	B
Lena	99.62	99.61	99.60	33.46	33.47	33.55
Fruits	99.60	99.61	99.62	33.47	33.48	33.47
Baboon	99.61	99.63	99.61	33.46	33.48	33.45
Peppers	99.60	99.62	99.60	33.40	33.43	33.52
Airplane	99.60	99.61	99.62	33.47	33.43	33.46
Monarch	99.61	99.60	99.62	33.50	33.49	33.44
Girl	99.61	99.61	99.61	33.41	33.51	33.48

**Table 8:** The NPCR and UACI values for the plain image sensitivity of all tested encryption methods on Lena.

Methods	NPCR(%)			UACI(%)		
	R	G	B	R	G	B
The Proposed	99.62	99.61	99.60	33.46	33.47	33.55
Ref. [28]	99.62	99.62	99.62	33.47	33.43	33.47
Ref. [27]	99.61	99.63	99.61	33.45	33.46	33.45
Ref. [35]	99.60	99.58	99.59	33.44	33.43	33.43
Ref. [20]	99.58	99.57	99.58	33.34	33.34	33.34
Ref. [31]	99.59	99.60	99.59	33.33	33.33	33.33

**Table 9:** The values of information entropies for the cipher images provided by the proposed encryption approach.

Images	R	G	B
Lena	7.9993	7.9994	7.9993
Fruits	7.9993	7.9994	7.9993
Baboon	7.9993	7.9993	7.9994
Peppers	7.9993	7.9993	7.9994
Airplane	7.9993	7.9993	7.9993
Monarch	7.9995	7.9995	7.9995
Girl	7.9995	7.9995	7.9996

**Table 10:** The information entropies of all components in the encryption results of Lena.

Methods	R	G	B
The Proposed	7.9993	7.9994	7.9993
Ref. [28]	7.9993	7.9992	7.9994
Ref. [27]	7.9992	7.9992	7.9992
Ref. [35]	7.9992	7.9993	7.9994
Ref. [20]	7.9992	7.9992	7.9992
Ref. [31]	7.9976	7.9976	7.9976

**Table 11:** A comparison of several encryption methods on the PSNR values of all benchmark images.

Images	The Proposed	Ref. [28]	Ref. [31]	Ref. [20]	Ref. [27]	Ref. [48]	Ref. [47]
Lena	83.022	83.022	80.927	81.131	83.021	81.225	81.027
Airplane	83.022	83.022	82.234	82.462	83.022	82.531	82.478
Fruits	83.022	83.022	80.835	81.023	83.023	80.632	81.147
Peppers	83.022	83.022	83.073	82.971	83.021	83.105	82.735
Baboon	83.022	83.022	83.145	82.873	83.023	82.534	82.652
Girl	84.783	84.783	84.641	84.653	84.782	84.685	84.437
Monarch	84.783	84.783	84.697	84.732	84.784	84.746	84.625

**Table 12:** The statistical data obtained for the encryption results provided by all tested methods on the variances of histograms.

Components		The Proposed	Ref. [20]	Ref. [28]	Ref. [27]	Ref. [31]	Ref. [35]
R	Mean	609.98	637.28	594.79	583.19	625.53	648.91
	STD	46.72	46.76	53.73	44.21	45.94	47.52
G	Mean	588.31	646.37	601.27	586.32	631.13	644.93
	STD	36.30	52.94	49.06	50.37	54.06	55.28
B	Mean	598.01	663.71	617.94	607.25	656.22	666.21
	STD	34.87	96.92	45.06	89.61	95.66	98.91

**Table 13:** The statistical data on the information entropies of the encryption results provided by all tested methods.

Methods	R		G		B	
	Mean	STD	Mean	STD	Mean	STD
The Proposed	7.9988	0.0001	7.9988	0.0001	7.9988	0.0001
Ref. [28]	7.9988	0.0001	7.9988	0.0001	7.9988	0.0001
Ref. [27]	7.9961	0.0005	7.9962	0.0006	7.9962	0.0005
Ref. [35]	7.9952	0.0004	7.9953	0.0003	7.9954	0.0004
Ref. [20]	7.9947	0.0005	7.9982	0.0004	7.9952	0.0004
Ref. [31]	7.9935	0.0003	7.9936	0.0002	7.9935	0.0003

**Table 14:** The statistical data on the PSNRs of the encryption results generated by all tested encryption approaches.

Methods	PSNR	
	Mean	STD
The Proposed	80.7231	0.0000
Ref. [28]	80.7231	0.0000
Ref. [27]	80.7229	0.0001
Ref. [35]	80.6343	0.0002
Ref. [20]	80.4352	0.0001
Ref. [31]	80.2396	0.0002

**Table 15:** The statistical data on the key sensitivity NPCRs and UACIs in the encryption results generated by all tested methods.

Methods		NPCR (%)			UACI (%)		
		R	G	B	R	G	B
The Proposed	Mean	99.6055	99.6060	99.6105	33.4490	33.4780	33.4645
	STD	0.0157	0.0150	0.0193	0.0547	0.0573	0.0610
Ref. [28]	Mean	99.6025	99.6110	99.6130	33.4700	33.4835	33.4555
	STD	0.0152	0.0155	0.0130	0.0439	0.0589	0.0867
Ref. [27]	Mean	99.6233	99.6231	99.6232	33.4512	33.4615	33.4513
	STD	0.0221	0.0232	0.0124	0.1053	0.0733	0.1042
Ref. [35]	Mean	99.6002	99.6003	99.6006	33.4315	33.4326	33.4378
	STD	0.0204	0.0213	0.0193	0.1032	0.0985	0.0927
Ref. [20]	Mean	99.6203	99.6201	99.6202	33.4432	33.4428	33.4527
	STD	0.0225	0.0213	0.0204	0.0923	0.1121	0.1027
Ref. [31]	Mean	99.6152	99.6151	99.6153	33.4302	33.4301	33.4415
	STD	0.0227	0.0208	0.0183	0.0925	0.0834	0.1023

**Table 16:** The statistical data on the plain image sensitivity NPCRs and UACIs for the R, G, and B components in the cipher images generated with all tested methods.

Methods		NPCR (%)			UACI (%)		
		R	G	B	R	G	B
The Proposed	Mean	99.6068	99.6121	99.6116	33.4653	33.4595	33.4421
	STD	0.0153	0.0147	0.0125	0.0375	0.0695	0.0458
Ref. [28]	Mean	99.6033	99.6121	99.6107	33.4642	33.4527	33.4658
	STD	0.0101	0.0098	0.0125	0.1017	0.1032	0.1073
Ref. [27]	Mean	99.6062	99.6053	99.6044	33.4573	33.4582	33.4535
	STD	0.0032	0.0011	0.0023	0.1124	0.1025	0.1327
Ref. [35]	Mean	99.5842	99.5837	99.5842	33.4435	33.4483	33.4216
	STD	0.0026	0.0034	0.0015	0.1127	0.1103	0.1114
Ref. [20]	Mean	99.5873	99.5852	99.5973	33.4592	33.4519	33.4612
	STD	0.0015	0.0026	0.0031	0.0833	0.1015	0.0954
Ref. [31]	Mean	99.5851	99.5842	99.5862	33.4532	33.4518	33.4526
	STD	0.0023	0.0021	0.0017	0.0903	0.0874	0.1217

## Data Availability

The data in this study is available at the following link https://github.com/yinglei2020/image-encrypt-data, accessed on 9 May 2024.

## References

[1] Alvarez G., Li S.J. (2006). Some basic cryptographic requirements for chaos-based cryptosystem. Int. J. Bifurcat. Chaos.

[2] Kanso A., Ghebleh M. (2017). An algorithm for encryption of secret images into meaningful images. Opt. Lasers Eng..

[3] Zhang G., Liu Q. (2011). A novel image encryption method based on total shuffling scheme. Opt. Commun..

[4] Liu Z., Xu L., Lin C., Dai J., Liu S. (2011). Image encryption scheme by using iterative random phase encoding in gyrator transform domains. Opt. Lasers Eng..

[5] Wang Y., Wong K.W., Liao X., Chen G. (2011). A new chaos-based fast image encryption algorithm. Appl. Soft Comput. J..

[6] Huang C.K., Nien H.H. (2009). Multi chaotic systems based pixel shuffle for image encryption. Opt. Commun..

[7] Chen G., Mao Y., Chui C.K. (2004). A symmetric image encryption scheme based on 3D chaotic cat maps. Chaos Solitons Fractals.

[8] Zhu Z.L., Zhang W., Wong K.W., Yu H. (2011). A chaos based symmetric image encryption scheme using a bit-level permutation. Inf. Sci..

[9] Guo Q., Liu Z., Liu S. (2010). Color image encryption by using Arnold and discrete fractional random transforms in IHS space. Opt. Lasers Eng..

[10] Tao R., Meng X.Y., Wang Y. (2010). Image encryption with multiorders of fractional fourier transforms. IEEE Trans. Inf. Forensics Secur..

[11] Wang X.Y., Yang L., Liu R., Kadir A. (2010). A chaotic image encryption algorithm based on perceptron model. Nonlinear Dyn..

[12] Zhao X.Y., Chen G. Ergodic matrix in image encryption. Proceedings of the 2nd International Conference on Image and Graphics.

[13] Zunino R. (1998). Fractal circuit layout for spatial decorrelation of images. Electron. Lett..

[14] Cao W., Zhou Y., Chen C.L.P., Xia L. (2017). Medical image encryption using edge maps. Signal Process..

[15] Toughi S., Fathi M.H., Sekhavat Y.A. (2017). An image encryption scheme based on elliptic curve pseudo random and advanced encryption system. Signal Process..

[16] Liu Z., Chen H., Liu T., Li P., Xu L., Dai J., Liu S. (1993). Image encryption by using gyrator transform and Arnold transform. J. Electron. Imaging.

[17] Kumar M., Iqbal A., Kumar P. (2016). A new RGB image encryption algorithm based on DNA encoding and elliptic curve Diffie-Hellman cryptography. Signal Process..

[18] Ye G., Pan C., Dong Y., Shi Y., Huang X. (2020). Image encryption and hiding algorithm based on compressive sensing and random numbers insertion. Signal Process..

[19] Mirzaei M.Y., Irani H. (2012). A new image encryption method: Parallel sub–image encryption with hyper chaos. Nonlinear Dyn..

[20] Jithin K.C., Sankar S. (2020). Colour image encryption algorithm combining Arnold map, DNA sequence operation, and a Mandelbrot set. J. Inf. Secur. Appl..

[21] Zahid A.H., Al-Solami E., Ahmad M. (2020). A Novel Modular Approach Based Substitution-Box Design for Image Encryption. IEEE Access.

[22] Chen J., Zhu Z., Zhang L., Zhang Y., Yang B. (2018). Exploiting self-adaptive permutation-diffusion and DNA random encoding for secure and efficient image encryption. Signal Process..

[23] Bao W., Zhu C. (2022). A secure and robust image encryption algorithm based on compressive sensing and DNA coding. Multim. Tools Appl..

[24] Liu H., Wang X. (2010). Color image encryption based on one-time keys and robust chaotic maps. Comput. Math. Appl..

[25] Lu Q., Yu L., Zhu C. (2022). Symmetric Image Encryption Algorithm Based on a New Product Trigonometric Chaotic Map. Symmetry.

[26] Song Y., Song J., Qu J. A secure image encryption algorithm based on multiple one-dimensional chaotic systems. Proceedings of the 2016 2nd IEEE International Conference on Computer and Communications.

[27] Adeel M., Song Y. (2022). Secure Encryption of Color Images with Chaotic Systems and Particle Swarm Optimization. Iran J. Sci. Technol. Trans. Electr. Eng..

[28] Man X., Song Y. (2023). Encryption of Color Images with an evolutionary framework controlled by chaotic systems. Entropy.

[29] Wang X.Y., Zhang Y.Q., Bao X.M. (2015). A novel chaotic image encrypytion scheme using DNA sequence operations. Opt. Lasers Eng..

[30] Hu T., Liu Y., Gong L.H., Guo S.F., Yuan H.M. (2017). Chaotic image cryptosystem using DNA deletion and DNA insertion. Signal Process.

[31] Liu L., Wang D., Lei Y. (2020). An image encryption scheme based on hyper chaotic system and DNA with fixed secret keys. IEEE Access.

[32] Xian Y., Wang X. (2021). Fractal sorting matrix and its application on chaotic image encryption. Inf. Sci..

[33] Hua Z., Zhu Z., Yi S., Zhang Z., Huang H. (2021). Cross-plane colour image encryption using a two-dimensional logistic tent modular map. Inf. Sci..

[34] Guesmi R., Farah M.A.B., Kachouri A., Samet M. (2016). A novel chaos-based image encryption using DNA sequence operation and secure hash algorithm SHA-2. Nonlinear Dyn..

[35] Cai S., Huang L., Chen X., Xiong X. (2018). A symmetric plaintext-related color image encryption system based on bit permutation. Entropy.

[36] Zhang Q., Guo L., Wei X. (2013). A novel image fusion encryption algorithm based on DNA sequence operation and hyper-chaotic system. Optik.

[37] Zhang Y.Q., Wang X.Y., Liu J., Chi Z.L. (2016). An image encryption scheme based on the MLNCML system using DNA sequences. Opt. Lasers Eng..

[38] Li X., Wang L., Yan Y., Liu P. (2016). An improvement color image encryption algorithm based on DNA operations and real and complex chaotic systems. Optik.

[39] Belazi A., EI-Latif A.A.A., Belghith S. (2016). A novel image encryption scheme based on substitution-permutation network and chaos. Signal Process..

[40] Arbelaez P., Maire M., Fowlkes C., Malik J. (2011). Contour detection and hierarchical image segmentation. IEEE Trans. Pattern Anal. Mach. Intell..

[41] Edward O. (2003). Chaos in Dynamical Systems.

[42] Pak C., Huang L. (2017). A new color image encryption using combination of the 1d chaotic map. Signal Process..

[43] Fu C., Huang J.B., Wang N.N., Hou Q.B., Lei W.M. (2014). A symmetric chaos-based image cipher with an improved bit-level permutation strategy. Entropy.

[44] Ganesan K., Murali K. (2014). Image encryption using eight dimensional chaotic cat map. Eur. Phys. J. Spec. Top..

[45] Murillo-Escobar M.A., Cruz-Hernández C., Abundiz-Pérez F., López-Gutiérrez R.M., Campo O.R.A.D. (2015). A RGB image encryption algorithm based on total plain image characteristics and chaos. Signal Process..

[46] Mollaeefar M., Sharif A., Nazari M. (2015). A novel encryption scheme for colored image based on high level chaotic maps. Signal Process..

[47] Li C., Luo G., Qin K., Li C. (2017). An image encryption scheme based on chaotic tent map. Nonlinear Dyn..

[48] Wu J., Liao X., Yang B. (2018). Image encryption using 2D Hénon-sine map and DNA approach. Signal Process..

[49] Yue W., Noonan J.P., Agaian S. (2011). NPCR and UACI randomness tests for image encryption. Cyber J..

[50] Zhu S., Deng X., Zhang W., Zhu C. (2023). Secure image encryption scheme based on a new robust chaotic map and strong S-box. Math. Comput. Simul..

[51] Li C.L., Zhou Y., Li H.M., Feng W., Du J.R. (2021). Image encryption scheme with bit-level scrambling and multiplication diffusion. Multimed. Tools Appl..

[52] Wen H., Lin Y., Feng Z. (2024). Cryptanalyzing a bit-level image encryption algorithm based on chaotic maps. Eng. Sci. Technol. Int. J..

